# Elucidating a Complicated Enantioselective Metabolic Profile: A Study From Rats to Humans Using Optically Pure Doxazosin

**DOI:** 10.3389/fphar.2022.834897

**Published:** 2022-03-10

**Authors:** Dezhi Kong, Yuan Tian, Kunfeng Duan, Wenyan Guo, Qingning Zhang, Panpan Zhang, Zuxiao Yang, Xia Qin, Leiming Ren, Wei Zhang

**Affiliations:** ^1^ Department of Pharmacology of Chinese Materia Medica, School of Chinese Integrative Medicine, Hebei Medical University, Shijiazhuang, China; ^2^ Department of Pharmacy, Third Hospital of Hebei Medical University, Shijiazhuang, China

**Keywords:** pure optical isomers, enantioselectivity, doxazosin, liver microsomes, cytochrome P450 enzymes, comprehensive metabolic system

## Abstract

Doxazosin (DOX) is prescribed as a racemic drug for the clinical treatment of benign prostatic hyperplasia and hypertension. Recent studies found that the two enantiomers of DOX exhibit differences in blood concentration and pharmacological effects. However, the stereoselective metabolic characteristics and mechanisms for DOX are not yet clear. Herein, we identified 34 metabolites of DOX in rats based on our comprehensive and effective strategy. The relationship among the metabolites and the most discriminative metabolites between (−)-DOX and (+)-DOX administration was analyzed according to the kinetic parameters using state-of-the-art multivariate statistical methods. To elucidate the enantioselective metabolic profile *in vivo* and *in vitro*, we carefully investigated the metabolic characteristics of metabolites after optically pure isomers administration in rat plasma, rat liver microsomes (RLMs) or human liver microsomes (HLMs), and recombinant human cytochrome P450 (CYP) enzymes. As a result, the differences of these metabolites were found based on their exposure and elimination rate, and the metabolic profile of (±)-DOX was more similar to that of (+)-DOX. Though the metabolites identified in RLMs and HLMs were the same, the metabolic profiles of the metabolites from (−)-DOX and (+)-DOX were greatly different. Furthermore, four human CYP enzymes could catalyze DOX to produce metabolites, but their preferences seemed different. For example, CYP3A4 highly specifically and selectively catalyzed the formation of the specific metabolite (M22) from (−)-DOX. In conclusion, we established a comprehensive metabolic system using pure optical isomers from *in vivo* to *in vitro*, and the complicated enantioselectivity of the metabolites of DOX was clearly shown. More importantly, the comprehensive metabolic system is also suitable to investigate other chiral drugs.

## Introduction

Doxazosin (DOX), a long-acting and highly selective α_1_ receptor blocker, is commonly used in treating benign prostatic hyperplasia (BEH) as the first-line therapy. It can relax the smooth muscle of the prostate and relieve the lower urinary tract symptoms related to BEH by targeting the α_1_-receptor in the prostate tissue, especially near the bladder neck (Cao et al., 2016; Fusco et al., 2016). DOX is also an additive drug for clinical antihypertensive treatment (Williams et al., 2015), especially suitable for elderly patients with BEH accompanied by hypertension. Recently, it has been described that DOX have anti-tumor effects by inhibiting cell proliferation, arresting cell cycle, and inducing apoptosis (Ramírez-Expósito and Martínez-Martos, 2019; Wade et al., 2019; Suzuki K. et al., 2020; Karaca et al., 2021). Nowadays, the prescribed drug of DOX is a racemic drug [(±)-DOX] composed of equal mixtures of (−)-DOX and (+)-DOX. However, differences in pharmacological effects between (−)-DOX and (+)-DOX have been reported; that is, the blocking effect of (−)-DOX on α_1D_ receptors in a rat vascular smooth muscle is weaker than that of (+)-DOX. They produce opposite inotropic effects in the rat atria, and the chiral carbon atom in the molecular structure of doxazosin does not affect its activity at the therapeutic target of α_1A_ receptors in the rabbit prostate (Zhao et al., 2012). Undoubtedly, the enantioselective pharmacodynamics is related to the difference of enantiomers in pharmacokinetics. Therefore, elucidating the pharmacokinetics of (−)-DOX, (+)-DOX, and (±)-DOX has become an important issue for DOX clinical application.

Actually, we have reported the differences in pharmacokinetics between (−)-DOX and (+)-DOX (Liu et al., 2010; Zhen et al., 2013; Li et al., 2015; Kapri et al., 2019). The plasma concentration ratio of (+)-DOX to (−)-DOX (C_(+)-DOX/(-)DOX_) is increased from 1.7 at 10 min to 17.1 at 360 min after a single injection of (±)-DOX into rat tail vein (Li et al., 2015). Moreover, the elimination of (+)-DOX was slower than that of (−)-DOX in rat liver microsomal system during incubation with (±)-DOX (Kong et al., 2015). The stereoselectivity of enzymes participating in drug metabolism is a well-known knowledge. For example, L-nebivolol was reported to be highly metabolized by CYP2D6, but CYP2C19 was the primary enzyme responsible for D-nebivolol (Lefebvre et al., 2007; Kelley et al., 2019). CYP2D6 preferentially metabolizes (−)-tramadol to (−)-O-desmethyltramadol rather than (+)-tramadol to (+)-O-desmethyltramadol (Suzuki S. et al., 2020). Though we found that CYP3A might be involved in the chiral metabolism of DOX in rats (Kong et al., 2015), a deeper understanding of the stereoselective metabolism is still poor due to the lack of knowledge regarding the metabolites of DOX and its metabolic pathways.

In recent years, liquid chromatography coupled with mass spectrometry (LC-MS) has emerged as one of the most powerful analytical tools for the screening and identifying drug metabolites with low-nanomolar sensitivity and high specificity (Pang et al., 2019; Higashi and Ogawa, 2020). Moreover, the Orbitrap mass detector with up to six orders of linear dynamic range in a high-resolution acquisition mode is beneficial to improving the simultaneous quantification of doxazosin and its metabolites. In our preliminary experiments, we found 98 potential metabolites identified from the rat plasma after intravenous administration of (±)-DOX. Herein, we further plan to confirm the metabolites with chemical structures for DOX and elucidate the stereoselective metabolic characteristics and mechanisms by analyzing the kinetic properties of metabolites after optically pure isomers administration in rat plasma, rat liver microsomes (RLMs) or human liver microsomes (HLMs), and seven recombinant human cytochrome P450 (CYP) enzymes.

## Materials and Method

### Chemicals and Biological Reagents

(±)-Doxazosin mesylate [(±)-DOX], (+)-doxazosin mesylate [(+)-DOX], and (−)-doxazosin mesylate [(−)-DOX] standards (>99.9% purity) were provided by the New Drug Research and Development Center of the North China Pharmaceutical Group Corporation (Shijiazhuang, China). β-Nicotinamide adenine dinucleotide phosphate hydrate (NADP), glucose-6-phosphate dehydrogenase from Baker’s yeast (*S. cerevisiae*), glucose-6-phosphate, prazosin (internal standard, IS), phenacetin, tolbutamide, and human CYP enzymes (CYP3A4, CYP2D6, CYP2C19, CYP2C8, CYP1A2, CYP2E1, and CYP2C9) were purchased from Sigma-Aldrich (St Louis, USA). Dextromethorphan hydrobromide, paclitaxel, and testosterone were provided by TCI Shanghai (Shanghai, China). Chlorzoxazone and omeprazole were purchased from J&K Chemical (Beijing, China). Heparin sodium injection was provided by the Jiangsu Wanbang Biochemical Pharmaceutical Group Co., Ltd. (Xuzhou, China). Human and rat liver microsomes were purchased from BD Gentest (Franklin, USA). Tris base and MgCl_2_ were purchased from Tianjin Yongda Chemical Reagent Co., Ltd. (Tianjin, China). Ultrapure water was prepared by Thermo Scientific Nanopure Water Purifier (Waltham, USA). HPLC-grade methanol and acetonitrile were purchased from Thermo Fisher Scientific (Ottawa, ON, Canada). All the other chemicals were of analytical grade.

### Animals

Specific pathogen-free healthy male SD rats (180–200 g) were purchased from the Vital River Laboratory Animal Company (Beijing, China; certificate no. SCXK 2016-0006). All rats were fed with standard guidelines and housed in well-ventilated cages at room temperature (23 ± 2°C) with a regular 12 h light-dark cycle. The rats were allowed free access to commercial aseptic food and pure water before the experiment. This study was approved by the Animal Ethics Committee of Hebei Medical University, complying with the National Research Council’s Guide for the Care and Use of Laboratory Animals (Approval no. IACUC-Hebmu-2021017).

### Quantitation of the Content of (+)-DOX, (−)-DOX, or (±)-DOX in Pure Form

To keep the same dosages for (+)-DOX, (−)-DOX, or (±)-DOX administration, (+)-DOX, (−)-DOX, or (±)-DOX were analyzed on the achiral C18 column by an Agilent 1260 HPLC system coupled with fluorescence detector according to our previous report (Zhen et al., 2013). Isocratic elution was conducted using a mobile phase of phosphate buffer-acetonitrile (85: 15, *v/v*) at a flow rate of 0.8 ml/min. The fluorescence detection was set at λ_Ex_ = 255 nm and λ_Em_ = 385 nm. The ratio of the peak areas of (+)-DOX, (−)-DOX, and (±)-DOX is 1.02: 1.22: 1. Furthermore, the following administrations for (+)-DOX, (−)-DOX, and (±)-DOX were adjusted according to the ratio.

### Administration and Sample Collection

The dosing and sampling procedures were similar to our former publication (Li et al., 2015). Briefly, 18 male rats were randomly divided into three groups (*n* = 6). The rats in each group received a single intravenous bolus injection of 6 mg/kg dose of (+)-DOX, (−)-DOX, or (±)-DOX without anesthesia, respectively. The blank blood samples were collected before administration, and blood samples were collected at 10, 30, 60, 90, 120, 240, 360, 480, and 600 min after drug administration. Samples were placed in heparinized centrifuge tubes and centrifuged at 2000 g for 10 min. The supernatants were collected and stored at −40°C until analysis.

### Incubation of Doxazosin With Liver Microsomes/Cytochrome P450s

For the metabolic study *in vitro*, rat liver microsomes, human liver microsomes, and seven recombinant human CYP enzymes (CYP3A4, CYPAD6, CYP2C19, CYP2C8, CYP1A2, CYP2E1, and CYP2C9) were used as metabolized enzymes. The concentration of the microsomal protein or each recombinant CYP enzyme in the incubation system was applied according to earlier studies (Kong et al., 2015; Kim et al., 2016). Briefly, the incubation system (400 µL) contained a microsomal protein (0.5 g/L) or CYP enzyme (40 nmol/L) and Tris-HCl buffer (100 mmol/L, pH 7.4) with MgCl_2_ (25 mmol/L). (−)-DOX, (+)-DOX, or (±)-DOX were the substrates with a concentration of 160 mg/ml. After 5 min of preincubation in the water bath at 37°C, reactions were initiated by adding 160 µL of NADPH-generating system (5.0 mM glucose-6-phosphate, 0.5 mM NADP^+^, 1 unit/ml glucose-6-phosphate dehydrogenase, and 5 mM MgCl_2_). The reactions were terminated after incubation for 0, 5, 10, 20, 30, 45, 60, and 80 min by adding three times the volume of ice-cold methanol containing prazosin (IS). Incubation without the addition of NADPH or DOX was used as a negative or positive control, respectively. Control experiments using boiled microsomes were also carried out. In the control samples, Tris-HCl buffer was added instead of the protein, NADPH or drug solution to ensure that the incubation volumes and compositions remained the same. All tests were performed in triplicate. The samples were prepared with the same method as the following “Sample preparation” procedures.

### Measurement of Recombinant Human CYP Enzymes Activity

The activity of each recombinant human CYP enzyme was evaluated by its CYP enzyme-specific substrate. The probe substrate for CYP3A4, CYP2D6, CYP2C19, CYP2C8, CYP1A2, CYP2E1, and CYP2C9 is testosterone, dextromethorphan, omeprazole, paclitaxel, phenacetin, chlorzoxazone, and tolbutamide, respectively. The final concentration of each substrate in the incubation system was 5 μmol/L. Other incubation conditions were the same as mentioned above, and the incubation duration was 30 min. 6β-Hydroxy testosterone metabolized by CYP3A4, dextrorphan by CYP2D6, 5-hydroxy omeprazole by CYP2C19, 6α-paclitaxel by CYP2C8, acetaminophen by CYP1A2, 6-hydroxy chlorzoxazone by CYP2E1, and 4′-hydroxytoluene butazone by CYP2C9 were determined using the UHPLC-HRMS system (Thermo Fisher Scientific, Waltham, MA, United States).

### Sample Preparation

Before analysis, samples were thawed and equilibrated at room temperature. Then, 100 µL of each plasma sample was transferred into a new centrifuge tube and spiked with three times the volume of methanol containing prazosin (IS). Each sample was vortexed to mix for 3 min and prepared by centrifugation at 12,000 g for 5 min at 4°C. The supernatant was removed to a new centrifugation tube and evaporated to dryness with a gentle stream of nitrogen in a 40°C water bath. The dry residue was dissolved with 100 µL of an acetonitrile-water mixture (1:1, *v/v*), vortexed for 3 min, and then centrifugated at 12,000 g for another 5 min. A five-microliter supernatant was injected into the UHPLC-HRMS system for analysis.

### UHPLC-HRMS Conditions

Thermo UltiMate 3000 UHPLC system coupled with Thermo Orbitrap Fusion high-resolution mass spectrometry (HRMS) detector with an H-ESI operating in a positive ion mode was used for all analyses. The chromatographic separations were performed on a Waters Xbridge C18 column (100 × 3 mm, 3.5 µm, Milford, MA, United States). The column temperature was maintained at 35°C. The mobile phases consisted of 0.2% ammonia solution (A) and acetonitrile (B) at a total flow rate of 0.5 ml/min. The total analysis time was 25 min, and a linear gradient condition was used as follows: 0–5 min, 10% B; 5–8 min, 10%–30% B; 8–17 min, 30%–70% B; 17–20 min, 70%–95% B; and 20–25 min, 95% B. Mass spectrometry conditions are as follows: sheath gas 40 Arb, aux gas 12 Arb, sweep gas 1 Arb, ion transfer tube temperature 330°C, and vaporizer temperature 317°C. MS^1^ and MS^2^ data were collected by Xcalibur software (Thermo Fisher Scientific, Waltham, MA, United States), acquiring as many MS^2^ data as possible within 0.6 s. A full scan was acquired in the range of 150–1,000 m/z at a resolution of 120,000 for the MS^1^ method, and the automatic gain control (AGC) was set at 2.0e^5^, RF-lens of 60%, and maximum injection time of 100 ms. The AGC was set at 5.0e^4^, and maximum ion injection times were 45 ms for MS^2^ scan. The activation type was high-energy collisional dissociation (HCD), and its energy was performed with 20%–40%. The dynamic exclusion duration time was set at 8 s.

Screening of the metabolites of DOX is shown by Gao et al. (2015), Vrobel et al. (2017), Bujak et al. (2020), and Izzo et al. (2020). In the analysis, data were processed with the Compound Discoverer (CD) 3.1 software and Mass Frontier 7.0 software (Thermo Fisher Scientific, Waltham, MA, United States). Raw files were imported into CD software to identify metabolites of DOX. The processing workflow “Find Expected metabolites with Fish Scoring and Background” was selected with the settings of mass tolerance, 5 ppm; intensity tolerance, 30%; minimum peak intensity, 100,000; ions (M + H)^+^, (M + K)^+^, and (M + Na)^+^; and RT tolerance, 0.3 min. The blank samples were used for the subtraction of the background compounds. The following filters were used: peak area >10,000; fish coverage >0; and no matches found in blank and solution samples. Considering the potential element compositions and the occurrence of possible reactions, the types and numbers of the predicted atoms were set as follows: C (0–35), H (0–50), O (0–25), S (0–2), N (0–7), and ring double bond (RDB) equivalent value (0–15). The maximum mass errors between the measured and the calculated values were fixed within 5 ppm. The detection of metabolites was accomplished *via* data processing with mass defect filtering (MDF) and control sample comparison. We also evaluated possible metabolites from isotopic ratios, peak shape, and fragments. The ClogP (Chemdraw Ultra 14.0, Cambridge Soft Corp., Cambridge, MA) was used to distinguish the structure of diastereoisomers containing two or more chiral centers because a diastereoisomer with a larger ClogP value had a longer retention time in reversed-phase liquid chromatography systems (Zhang et al., 2018). The possible structure of the metabolites was analyzed based on the MS^2^ data. Then, Mass Frontier 7.0 software (Thermo Fisher Scientific, Waltham, MA, United States) was used for further structure verification, which predicted the structures of a fragment based on the HighChem Fragmentation Library™ (Thermo Fisher Scientific, Waltham, MA, United States). The identified metabolites were added to our Local Compound Database for the following analysis to increase the chances of identifying the metabolites and reduce data processing time in a number of biological samples.

### Calculating the Kinetic Parameters for Metabolites

Chromatographic peaks were integrated by the TraceFinder software (Thermo Fisher Scientific, Waltham, MA, United States). The concentrations of DOX in plasma or incubation systems were determined using a calibration curve generated with the known concentrations. A comparison of the same metabolite among different samples was achieved by calculating the peak area ratio of the metabolite versus the internal standard at the MS^1^ level.

Pharmacokinetic parameters of (+)-DOX, (−)-DOX, and (±)-DOX and each metabolite were calculated based on concentration-time profiles of individual rat plasma samples using WinNonlin software (V.5.1, Pharsight, Mountain View, CA) by a noncompartmental analysis model. The maximum plasma concentration (C_max_) and the time to reach C_max_ (t_max_) were determined directly from the plot. The terminal phase rate constant (*λ*) was estimated as the absolute value of the slope of a linear regression during the apparent terminal phase of the natural logarithm transformed concentration-time profile. The area under the concentration-time curve (AUC) from time zero to the last quantifiable concentration (AUC_0-t_) was calculated using the linear trapezoidal method.

The kinetic parameters AUC_0-t_ for each metabolite in incubation experiments were also calculated using the linear trapezoidal method by WinNonlin software.

### Multivariate Statistical Analysis for the Metabolites Originating From (+)-DOX, (−)-DOX, and (±)-DOX

Multivariate data analysis was a powerful tool in the biological understanding and exploration of complex, multiparametric metabolic systems (Macherius et al., 2014; Commisso et al., 2017; Kim et al., 2018). Multivariate analyses were performed using the resulting matrix of lg(AUC) or lg(λ) of the observed metabolites in each sample. In this study, hierarchical cluster analysis (HCA), principal component analysis (PCA), and Pearson correlation analysis were employed to analyze the matrix by the RStudio platform (Version 1.0.143) installed R (Version 3.5.1) with packages such as ggplot2, procomp, corrgram, and heatmap.

### Other Statistical Analysis

All data were represented as mean ± standard deviation (mean ± SD). Logarithmic transformation was performed on AUC_0-t_ and C_max_ values before the statistical analysis. One-way ANOVA followed by Tukey’s HSD test by GraphPad Prism 5.0 (GraphPad Software, Inc., San Diego, CA) was used to analyze the difference in AUC_0-t_ or C_max_ among (−)-DOX, (+)-DOX, and (±)-DOX and that among the same metabolites of (−)-DOX, (+)-DOX, and (±)-DOX. *p*-value < 0.05 was considered statistically significant.

## Results and Discussion

### Identification and Structural Elucidation of Metabolites of Doxazosin

Up to now, no more than 10 known metabolites of DOX have been reported (Kaye et al., 1986). Therefore, a detailed study on the metabolites of DOX is necessary. For the identification of metabolites, (±)-DOX was administered to the rats through the caudal vein, and the blood was collected at different time points after administration and then analyzed by UHPLC-HRMS. To discover more metabolites of DOX, we used the method *in vivo* in the beginning rather than *in vitro* (such as microsomes or recombinant CYP enzymes) because there are many kinds of metabolic enzymes in the body, including carboxylases, dehydrogenases, lipoxygenases, oxidoreductases, kinases, lyases, and transferases (Alexander et al., 2011; Argikar et al., 2016)

**FIGURE 1:**
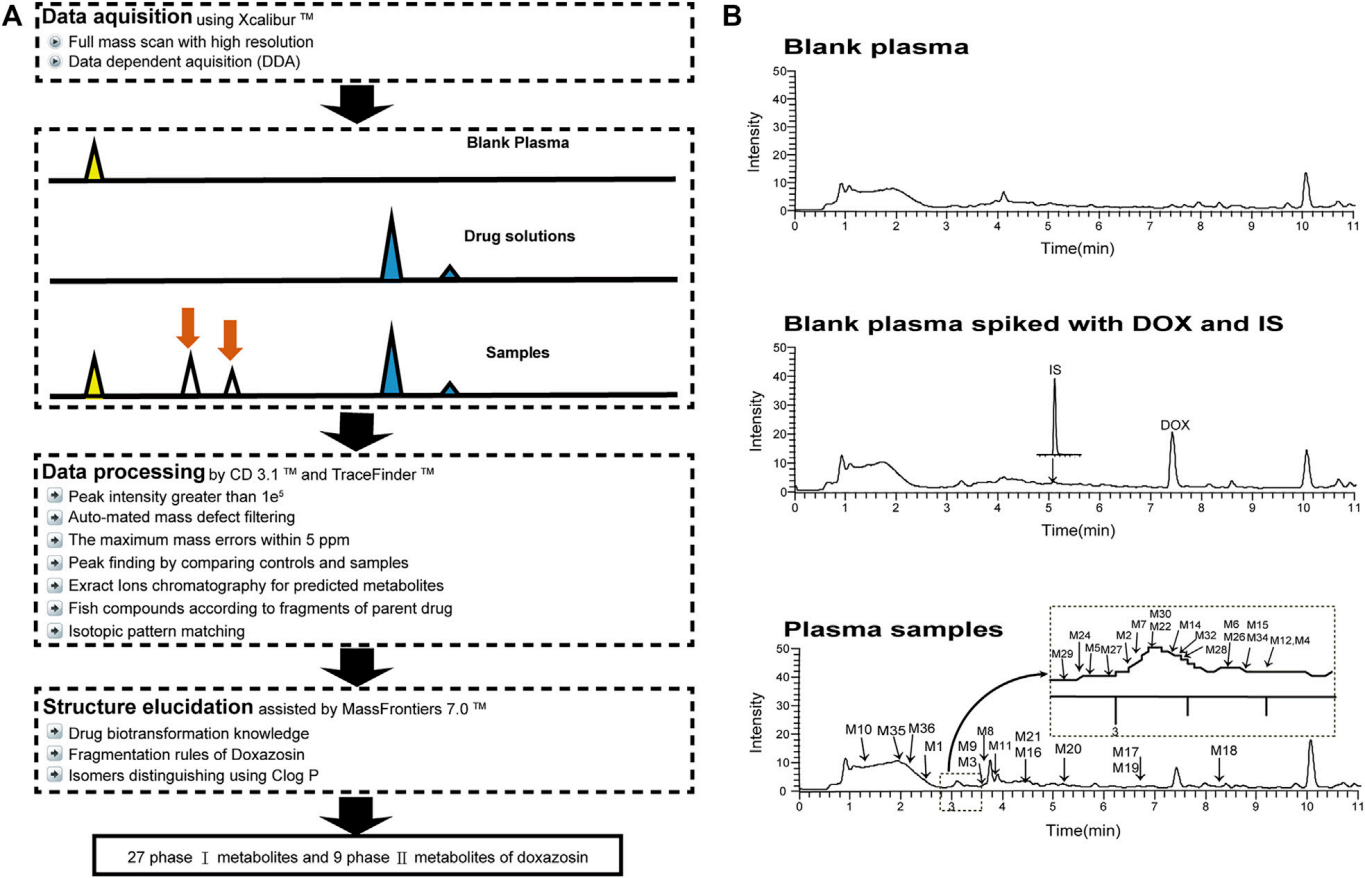
Analytical strategy for the detection and identification of DOX metabolites **(A)**, and the total ion chromatograms of blank and plasma samples after intravenous administration of DOX, indicating the 36 metabolites **(B)**.

In this study, we established a comprehensive and effective strategy (Figure 1A) to discover and identify the metabolites. Thirty-six metabolites were identified based on our strategy, and their possible chemical structures were inferred (Figure 1B and Table 1).

**TABLE 1:** Summary of 36 doxazosin metabolites with possible chemical structures.

Name	Formula	MS2: fragment ions	ΔMass	RT (min)	#MI	m/z	Identification
DOX	C23H25N5O5	344, 290, 247, 221	1.47	7.422	4	452,1935	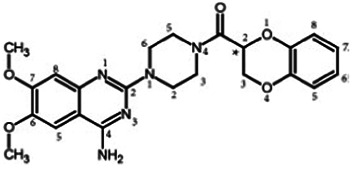
M1	C10H12N4O2	221, 206, 177	6.49	2.615	2	221,1047	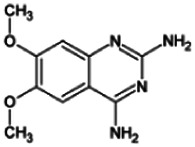
M2	C13H17N5O2	276, 247, 245, 221	1.79	3.03	3	276,1460	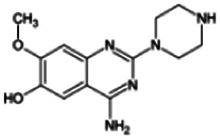
M3	C14H17N5O2	288, 245, 221, 170	7.12	3.089	2	288,1476	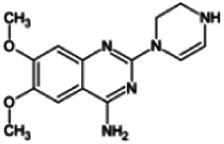
M4	C14H19N5O2	290, 247, 221	6.35	3.433	5	290,1630	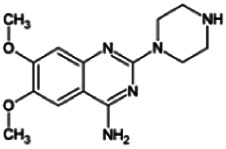
M5	C13H17N5O3	292, 274, 247	6.98	2.919	4	292,1425	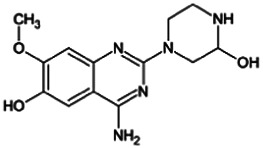
M6	C14H17N5O3	304, 276, 245, 221	1.39	3.328	2	304,1408	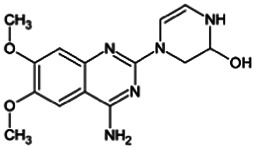
M7	C14H19N5O3	306, 288, 247, 221	1.70	3.056	2	306,1566	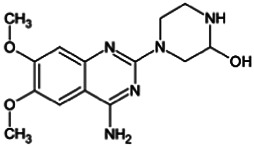
M8	C15H19N5O3	318, 290, 247	1.63	3.754	2	318,1566	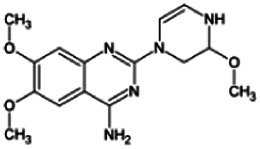
M9	C14H17N5O4	319, 263, 245	1.48	3.682	2	320,1358	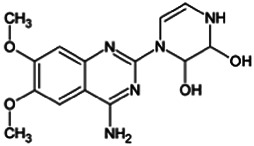
M10	C14H19N5O4	322, 247, 221	2.24	1.523	2	322,1517	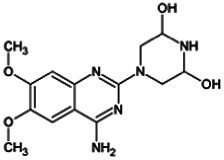
M11	C16H21N5O3	331, 290, 247	6.91	3.925	2	332,1740	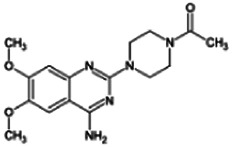
M12	C16H20N6O4	361, 290, 247	6.64	3.377	4	361,1643	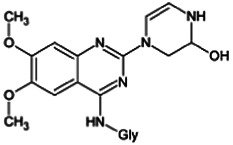
M13	C21H23N5O5	426, 408, 221	5.92	5.528	2	426,1797	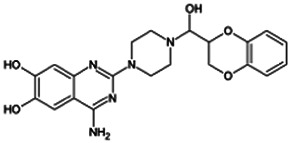
M14		426, 408, 300, 221	3.13	3.167	3	426,1785	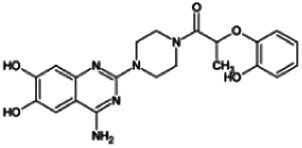
M15	C22H23N5O5	438, 330	1.86	3.331	2	438,1780	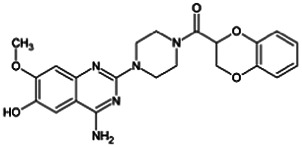
M16		438, 342, 287	1.72	4.279	2	438,1780	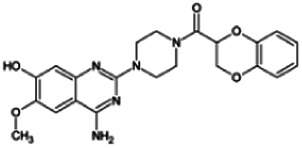
M17	C23H23N5O5	450, 342, 287, 245	6.62	6.565	4	450,1802	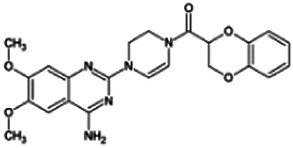
M18		450, 342, 287	4.79	8.322	3	450,1794	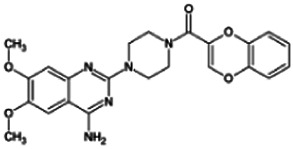
M19	C23H25N5O6	468, 450, 342, 247	6.55	6.594	3	468,1908	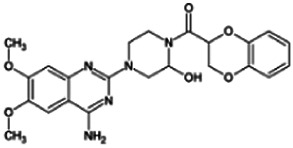
M21		468, 450, 290, 221	4.59	4.305	2	468,1899	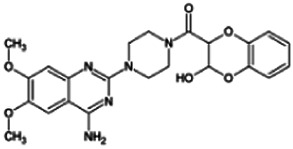
M22		468, 289, 221	6.42	3.091	4	468,1908	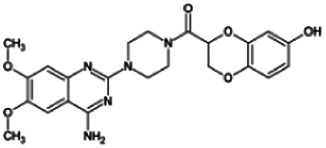
M20		468, 316, 290, 233	5.70	5.417	4	468,1904	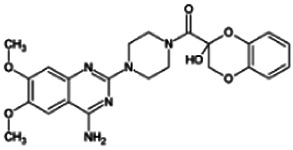
M23		468, 289, 221	6.22	2.861	2	468,1907	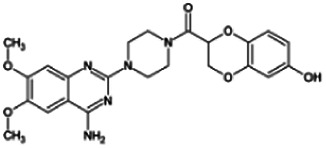
M24	C23H27N5O6	470, 360, 290, 233	7.57	2.877	3	470,2070	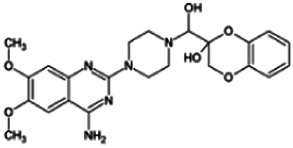
M25		470, 452, 142	7.38	5.023	2	470,2069	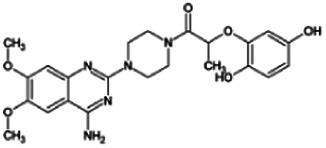
M26	C24H25N5O6	480, 318, 290, 135	6.13	3.37	4	480,1907	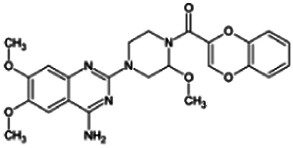
M27	C23H25N5O7	484, 440, 290, 233	6.5	2.973	3	484,1858	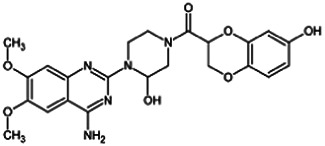
M28	C23H27N5O7	486, 468, 289, 221	5.92	3.189	4	486,2012	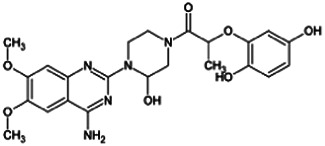
M29	C23H25N5O9S	548, 468, 344	6.60	2.908	3	548,1482	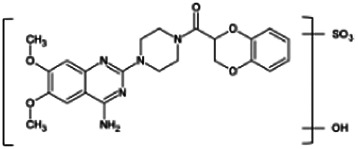
M30	C23H25N5O9S	548, 468, 344	6.65	3.081	2	548,1482	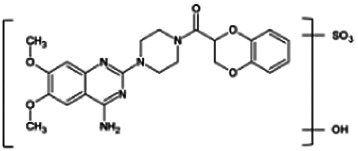
M31	C25H32N6O9S	592, 468, 221	6.22	3.073	4	593,2061	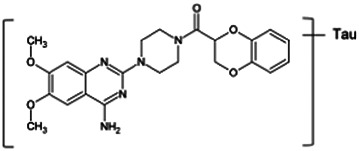
M32	C28H31N5O11	614, 438, 330	2.59	3.146	4	614,2109	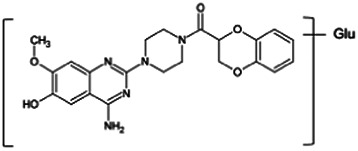
M33		614, 438	3.25	3.29	2	614,2109	
M34	C29H33N5O11	628, 452	2.40	3.35	4	628,2264	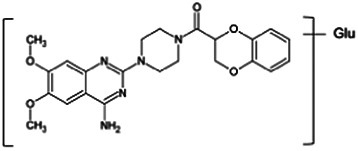
M35	C29H33N5O12	644, 468, 344	6.54	2.459	3	644,2241	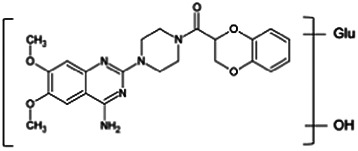
M36		644, 468, 344	6.82	2.538	4	644,2242	

**FIGURE 2:**
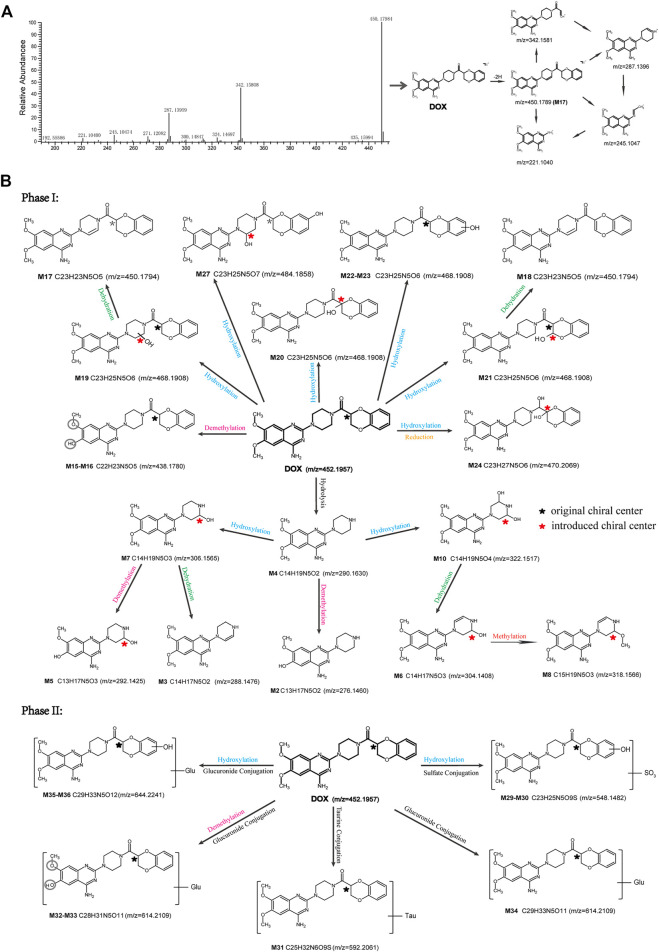
Elucidation of the chemical structure and mass fragmentation pattern of the metabolite taking M17 as an example **(A)**, and the proposed possible metabolic pathway for DOX **(B)**.

Firstly, the fragmentation patterns of DOX in ESI-HCD-MS were illustrated, which can provide useful information to deduce the structures of related metabolites. DOX showed a protonated (M + H)^+^ ion at m/z 452.1925 with a retention time of 7.4 min. It produced the base peak ion at m/z 344 [M + H–C_6_H_4_O_2_]^+^ in the ESI-HCD-MS^2^ spectrum. Because of the MS^3^ experiment, it is easy to know that the peak ion at m/z 290 was yielded from the ion at m/z 344 by losing C_3_H_2_O, and the other major characteristic ions at m/z 247 and 221 were yielded from the ion at m/z 290 by losing C_2_H_5_N and C_3_H_5_N_2_ (Supplementary Figure S1). Taking metabolite M17 as an example, it was eluted at 2.92 min and 2 Da (2H) less than DOX but had a similar characteristic fragment of m/z 221. Besides, the ions at m/z 245, m/z 288, and m/z 342 were also found by successively losing 2 Da of m/z 247, m/z 290, and m/z 344. Therefore, we inferred that the elimination reaction occurred at the sites of the piperazine ring, and the structure of M17 can be inferred easily (Figure 2A).

M1 (m/z 290.1628, C_10_H_12_N_4_O_2_) and M4 (m/z 290,1628, C_10_H_12_N_4_O_2_) were detected at 2.62 and 3.43 min, respectively. Because m/z 221.1040 and 290.1628 were the typical fragment ions of DOX and the fragment ions of M4 were m/z 247.1197 and 221.1038, we inferred M1 to be formed by quinazoline amino group cleavage and M4 to be formed by cleavage of a piperazine ring and a carbonyl linkage (Table 1 and Supplementary Figure S2). M2 with a protonated [M + H]^+^ ion at m/z 276.146 was eluted at 3.03 min, and its fragments of m/z 247.1197 and m/z 221.1038 were found in the mass spectrum of MS^2^ with no other characteristic ions observed. The molecular weight of M2 was 14 Da less than M4, showing a CH_2_ group loss. Therefore, it can be inferred that M2 is an O-demethyl metabolite of M4 (Table 1 and Supplementary Figure S2).

M19, M20, M21, M22, and M23 showed the same theoretical [M + H]^+^ ion at m/z 468,1899, which was 16 Da higher than that of DOX. Furthermore, they were eluted at 2.86, 3.1, 4.30, 5.41, and 6.59 min, respectively. It has been reported that DOX has monohydroxy metabolites (Kaye et al., 1986). Five monohydroxy metabolites were detected in our study (Table 1 and Supplementary Figure S2).

Elucidations for other metabolites are shown in detail in Supplementary Files (Supplementary Appendix S1, Table 1, and Supplementary Figure S2). The metabolites of DOX were grouped into phase I metabolites and phase II metabolites. Phase I metabolites included M1∼M11 and M13∼M28, and phase II metabolites included M12 and M29∼M36. According to the analysis of the structure of the metabolites, the possible metabolic pathways (Figure 2B) of DOX were proposed.

### Plasma Concentration-Time Profiles of the Metabolites of (−)-DOX, (+)-DOX, and (±)-DOX

The analysis methodology for DOX quantitation in plasma using LC-MS has been reported by our lab (Du et al., 2016). Owing to a lack of standard substances of DOX metabolites, M1∼M36 were semi-quantitatively analyzed by calculating peak area ratios of metabolites versus internal standard in extracted ion chromatograms. The method’s stability was evaluated using DOX, and the intra-day and inter-day coefficients of variation for the assay were less than 3.4% for DOX. The two enantiomers were stable during the entire course of the study, including the sample preparation, centrifugation, and the LC-MS assay.

**FIGURE 3:**
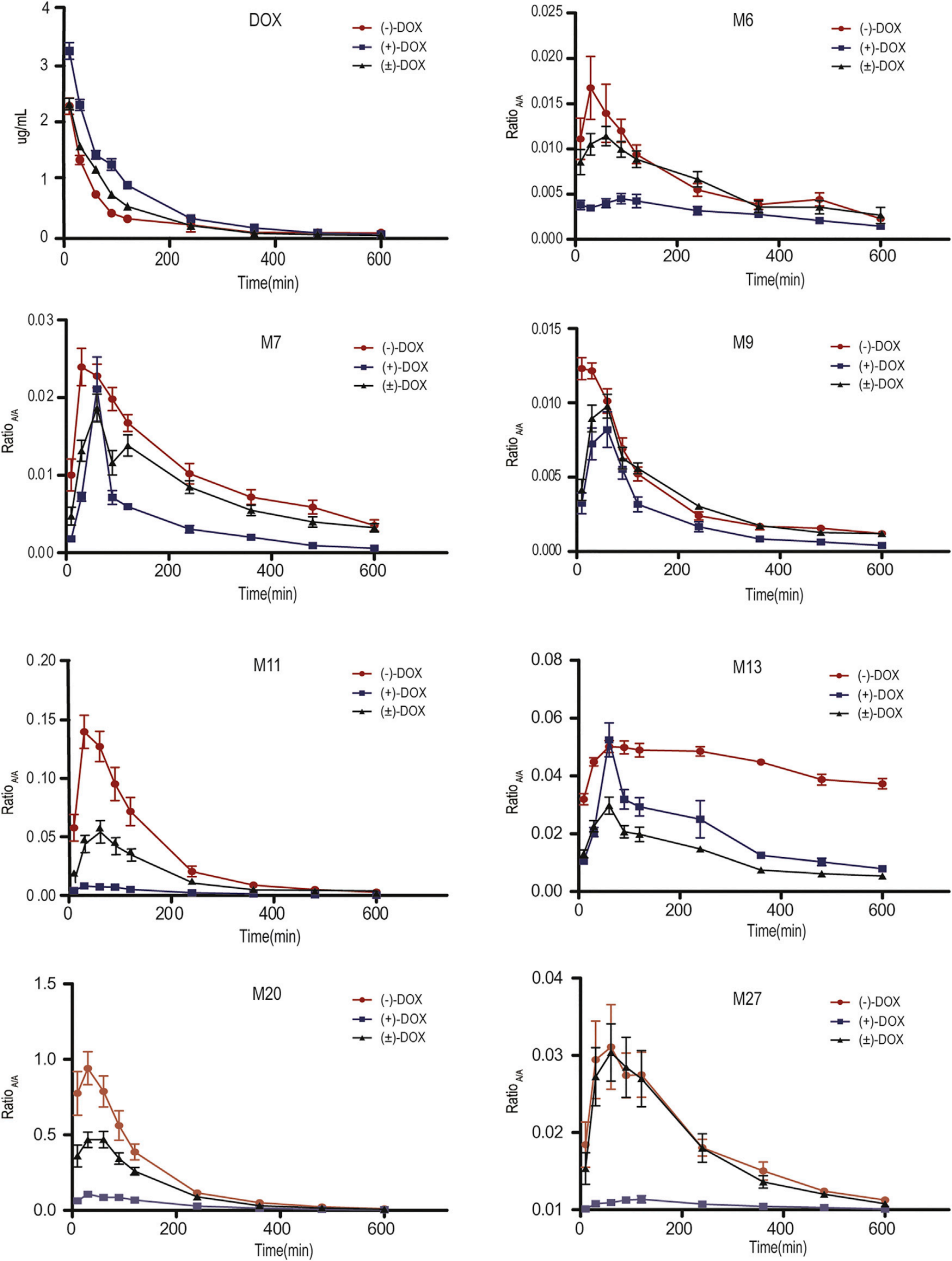
Plasma concentration-time curves for DOX and its representative metabolites obtained from six rats after (−)-DOX, (+)-DOX, or (±)-DOX administration.

Figure 3 and Supplementary Figure S3 show the mean plasma concentration-time curves for DOX and its metabolites obtained from six rats after intravenous injection of (−)-DOX, (+)-DOX, or (±)-DOX, respectively. The major pharmacokinetic parameters, including AUC_0-t_, C_max_, and λ, are summarized in Table 2, and the pharmacokinetic behaviors of (−)-DOX, (+)-DOX, and (±)-DOX were consistent with our previous report (Li et al., 2015).

**TABLE 2:** The pharmacokinetic parameters for doxazosin and its metabolites in six rats after (−)-DOX, (+)-DOX, or (±)-DOX administration (mean ± SD).

	AUC_0-t_			λ			C_max_		
	(−)-DOX	(+)-DOX	(±)-DOX	(−)-DOX	(+)-DOX	(±)-DOX	(−)-DOX	(+)-DOX	(±)-DOX
DOX	130.29 ± 35.42*^,+^	295.81 ± 50.87^#^	178.6 ± 11.97	0.015 ± 0.012	0.014 ± 0.004	0.013 ± 0.002	2.239 ± 0.357*	3.228 ± 0.365^#^	2.277 ± 0.252
M1	42.17 ± 8.81*	18.29 ± 3.89^#^	49.27 ± 11.05	—	0.002 ± 0.001	—	0.114 ± 0.037*	0.039 ± 0.008^#^	0.117 ± 0.026
M2	2.87 ± 0.43	2.73 ± 1.25	2.75 ± 0.56	0.002 ± 0.001*	0.006 ± 0.002^#^	0.003 ± 0.001	0.011 ± 0.004	0.026 ± 0.017	0.014 ± 0.003
M3	17.31 ± 3.23^+^	17.94 ± 5.25^#^	25.17 ± 2.96	0.002 ± 0.001	0.004 ± 0.002	0.004 ± 0.001	0.081 ± 0.025*	0.204 ± 0.095	0.117 ± 0.026
M4	493.78 ± 64.82*^,+^	257.79 ± 57.59^#^	359.55 ± 82.8	0.002 ± 0.001	0.003 ± 0.000	0.002 ± 0.001	2.195 ± 0.348	2.235 ± 1.071	1.369 ± 0.284
M5	17.03 ± 3.95	26.66 ± 11.73	21.17 ± 7.23	0.002 ± 0.001*^,+^	0.005 ± 0.001	0.005 ± 0.002	0.067 ± 0.028*	0.188 ± 0.081	0.134 ± 0.047
M6	3.85 ± 1.12*	1.76 ± 0.24^#^	3.51 ± 0.82	0.003 ± 0.001	0.004 ± 0.002	0.003 ± 0.001	0.017 ± 0.008*	0.006 ± 0.001^#^	0.012 ± 0.003
M7	6.29 ± 1.34*	2.23 ± 0.67^#^	4.69 ± 1.14	0.003 ± 0.001	0.006 ± 0.003	0.003 ± 0.001	0.025 ± 0.005	0.021 ± 0.01	0.019 ± 0.004
M8	2.38 ± 0.46*^,+^	1.12 ± 0.28	1.04 ± 0.22	0.005 ± 0.002	0.007 ± 0.002	0.005 ± 0.003	0.015 ± 0.001*^,+^	0.008 ± 0.003	0.005 ± 0.001
M9	2.14 ± 0.35*	1.28 ± 0.4^#^	1.99 ± 0.3	0.003 ± 0.001	0.004 ± 0.001	0.003 ± 0.001	0.013 ± 0.001*	0.009 ± 0.003	0.01 ± 0.002
M10	1.4 ± 0.3*^,+^	0.87 ± 0.12^#^	0.64 ± 0.12	0.002 ± 0.001	0.002 ± 0.001	0.002 ± 0.001	0.008 ± 0.002*^,+^	0.003 ± 0.001	0.003 ± 0.001
M11	20.68 ± 7.22*^,+^	1.56 ± 0.49^#^	9.95 ± 3.4	0.006 ± 0.002	0.008 ± 0.002	0.006 ± 0.001	0.14 ± 0.034*^,+^	0.008 ± 0.002^#^	0.058 ± 0.024
M12	6.25 ± 4.08*^,+^	33.56 ± 15.99	22.22 ± 8.13	0.016 ± 0.022	0.006 ± 0.001	0.007 ± 0.001	0.048 ± 0.032*^,+^	0.247 ± 0.111	0.161 ± 0.056
M13	26.33 ± 1.2*^,+^	11.59 ± 3.02^#^	7.47 ± 0.91	0.001 ± 0.000*^,+^	0.003 ± 0.000	0.003 ± 0.001	0.054 ± 0.004^+^	0.052 ± 0.014^#^	0.03 ± 0.007
M14	18.38 ± 2.7*^,+^	11.48 ± 4.56	8.55 ± 1.57	0.001 ± 0.001	0.001 ± 0.000	0.002 ± 0.001	0.06 ± 0.012*^,+^	0.026 ± 0.009	0.029 ± 0.01
M15	0.87 ± 0.39*^,+^	2.87 ± 0.79	2.74 ± 0.59	0.004 ± 0.002^+^	0.006 ± 0.001	0.007 ± 0.002	0.008 ± 0.004*^,+^	0.02 ± 0.005	0.02 ± 0.006
M16	1.00 ± 0.16	1.24 ± 0.32^#^	0.81 ± 0.12	0.002 ± 0.001	0.003 ± 0.002	0.004 ± 0.004	0.004 ± 0.001	0.005 ± 0.001^#^	0.003 ± 0.000
M17	70.22 ± 27.95*	128.25 ± 25.44	83.42 ± 13.15	0.008 ± 0.003	0.008 ± 0.001	0.008 ± 0.002	0.65 ± 0.185	0.774 ± 0.186	0.614 ± 0.112
M18	15.59 ± 4.00*^,+^	52.45 ± 14.19^#^	33.67 ± 5	0.005 ± 0.002	0.005 ± 0.001	0.006 ± 0.001	0.108 ± 0.023*^,+^	0.191 ± 0.041	0.148 ± 0.033
M19	18.75 ± 7.26*	28.16 ± 6.38	19.62 ± 3.01	0.008 ± 0.002	0.008 ± 0.001	0.009 ± 0.002	0.177 ± 0.049	0.223 ± 0.078	0.147 ± 0.026
M20	128.12 ± 36.76*^,+^	20.1 ± 2.45^#^	77.52 ± 19.63	0.007 ± 0.001	0.006 ± 0.001	0.007 ± 0.001	0.945 ± 0.271*^,+^	0.108 ± 0.023^#^	0.497 ± 0.141
M21	5.17 ± 0.77*^,+^	13.59 ± 1.92^#^	7.49 ± 1.8	0.004 ± 0.001*	0.007 ± 0.001	0.006 ± 0.001	0.048 ± 0.014*	0.123 ± 0.037^#^	0.054 ± 0.022
M22	82.59 ± 51.75*^,+^	390.78 ± 173.79	371.55 ± 134.21	0.006 ± 0.003	0.005 ± 0.001	0.007 ± 0.001	0.585 ± 0.358*^,+^	2.612 ± 1.213	2.502 ± 0.891
M24	5.88 ± 4.19*^,+^	21.95 ± 16.81	25.69 ± 9.48	0.010 ± 0.011	0.004 ± 0.001	0.005 ± 0.002	0.035 ± 0.023*^,+^	0.106 ± 0.072	0.15 ± 0.051
M25	10.87 ± 2.6	11.35 ± 2.9	12.07 ± 2.61	0.005 ± 0.001*^,+^	0.003 ± 0.001	0.004 ± 0.001	0.052 ± 0.015	0.04 ± 0.009	0.045 ± 0.011
M26	2.83 ± 1.53*^,+^	17.17 ± 8.39	10.92 ± 3.91	0.010 ± 0.006	0.004 ± 0.001	0.006 ± 0.002	0.032 ± 0.025*	0.252 ± 0.126	0.075 ± 0.035
M27	5.01 ± 2.21*	0.37 ± 0.28^#^	4.62 ± 2.33	0.006 ± 0.001	0.005 ± 0.003	0.007 ± 0.001	0.022 ± 0.013*	0.001 ± 0.001^#^	0.021 ± 0.01
M28	6.44 ± 4.28*^,+^	29.53 ± 12.76	31.85 ± 11.83	0.005 ± 0.002	0.005 ± 0.001	0.007 ± 0.001	0.045 ± 0.029*^,+^	0.201 ± 0.097	0.216 ± 0.079
M29	4.79 ± 0.71*^,+^	26.49 ± 5.58	27.13 ± 7.49	0.011 ± 0.003*^,+^	0.007 ± 0.000	0.007 ± 0.002	0.063 ± 0.017*^,+^	0.186 ± 0.028	0.197 ± 0.079
M31	3.66 ± 2.23*^,+^	17.7 ± 7.36	25.58 ± 8.37	0.007 ± 0.002	0.005 ± 0.001	0.006 ± 0.001	0.024 ± 0.013*^,+^	0.077 ± 0.03	0.162 ± 0.054
M32	4.3 ± 0.76*^,+^	25.25 ± 8.32	26.78 ± 6.04	0.004 ± 0.002*	0.001 ± 0.001	0.003 ± 0.001	0.003 ± 0.001*^,+^	0.014 ± 0.003^#^	0.032 ± 0.007
M33	0.41 ± 0.16*^,+^	5.98 ± 1.64	8.66 ± 1.65	0.004 ± 0.001*	0.001 ± 0.001^#^	0.003 ± 0.001	0.026 ± 0.003*^,+^	0.071 ± 0.012^#^	0.127 ± 0.033
M34	4.59 ± 0.9*^,+^	13.32 ± 4.18	14.08 ± 2.09	0.012 ± 0.002*^,+^	0.004 ± 0.000^#^	0.006 ± 0.001	0.034 ± 0.004^+^	0.038 ± 0.009^#^	0.064 ± 0.007
M35	2.47 ± 1.58	3.33 ± 0.92^#^	1.18 ± 0.31	0.010 ± 0.001*^,+^	0.006 ± 0.002	0.005 ± 0.003	0.023 ± 0.012^+^	0.015 ± 0.006^#^	0.008 ± 0.003
M36	6.51 ± 1.82*^,+^	18.37 ± 4.74	20.79 ± 6.72	0.003 ± 0.002	0.004 ± 0.001	0.004 ± 0.002	0.053 ± 0.011^+^	0.077 ± 0.018	0.097 ± 0.036

“-” means it cannot be calculated. Logarithmic transform was performed on AUC_0-t_ and C_max_ values before statistical analysis. One-way ANOVA followed by Tukey’s HSD test was used. *p < 0.05 versus (+)-DOX; ^+^ *p* < 0.05 versus (±)-DOX; ^#^ *p* < 0.05 versus (±)-DOX.

Thirty-six metabolites (M1∼M36) mentioned above were all detected in rat plasma after intravenous administration of (−)-DOX, (+)-DOX, or (±)-DOX. Because the concentration-time curves for M23 and M30 did not show a tendency, making it difficult to calculate their AUC values, other 34 metabolites were used as the available metabolites in the following data analysis.

The plasma exposure level of drug metabolites depends on the rank order of their ratio of formation to elimination kinetics (Brill et al., 2012). In the three parent agents, the plasma exposure of (+)-DOX was significantly higher, and that of (−)-DOX was lower than that of (±)-DOX, respectively (Figure 3). However, plasma exposure trends for M6, M7, M9, M11, M13, M20, and M27 were obviously different from their parent drugs. For example, the plasma exposure of M11 originating from (+)-DOX was much lower, and that from (−)-DOX was higher than that from (±)-DOX, respectively (Figure 3). Because the plasma exposure for other metabolites was very complicated and difficult to explain, it is necessary to employ a new statistical and data presentation method to reveal the secrets behind the data.

### The Complicated Relationship Among Metabolites

**FIGURE 4:**
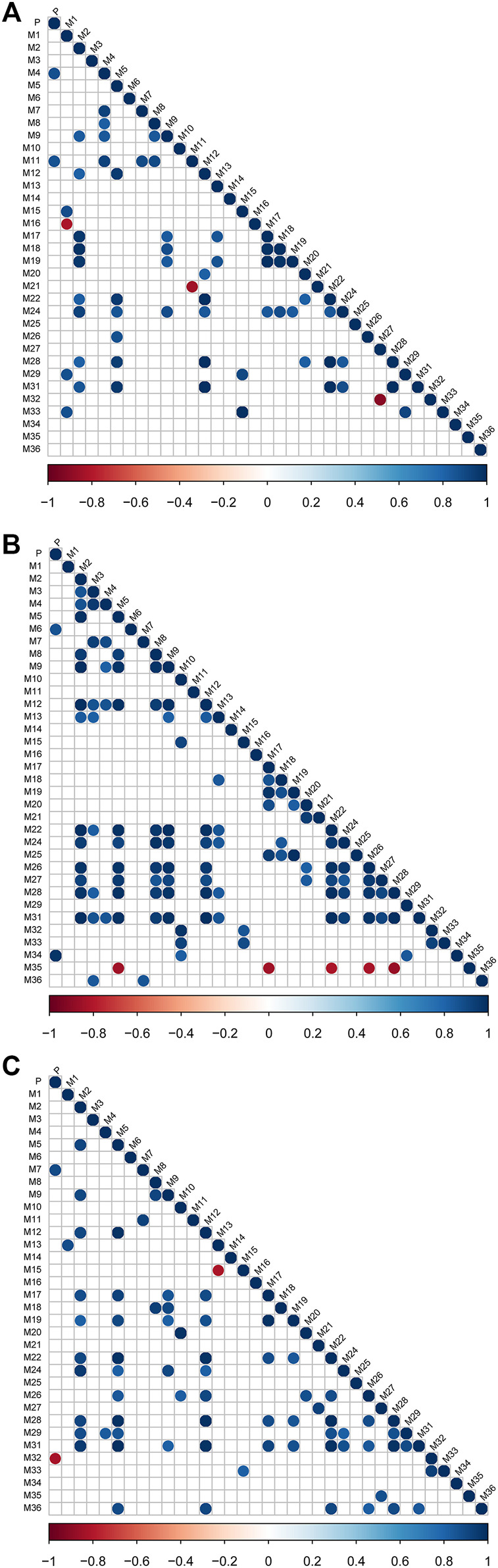
The correlation degree among metabolites after intravenous administration of (−)-DOX **(A)**, (+)-DOX **(B),** or (±)-DOX **(C)**. Horizontal and vertical axes represent correlation coefficients and 34 metabolites, respectively. Positive or negative correlations are displayed in blue or red, and color intensity is proportional to the correlation coefficient; that is, a darker color yields a stronger correlation (closer to −1 or 1). The insignificant correlations (*p* > 0.05) are blank.

Based on the AUC_0-t_ values of each metabolite, we evaluated the strength of the relationship among metabolites by the Pearson correlation analysis test. Figure 4 represents the correlations for all pairs of variables, indicating clear relationships among the metabolites of (−)-DOX, (+)-DOX, and (±)-DOX. Particularly, we found M2 positively associated with M9, M12, M22, M24, M28, and M31; M5 positively associated with M12, M22, M24, M26, M28, and M31; and M12 positively associated with M22, M24, M28, and M31 in all metabolites obtained from (−)-DOX, (+)-DOX, and (±)-DOX. Positive associations between M8 and M9, M15 and M33, and M17 and M19 were also revealed. This abundant information showed the possible upstream or downstream relationships of the metabolites, which further confirmed the possibility of the above metabolic pathways of DOX (Figure 2B).

### Revealing the Chiral Metabolism of DOX Using Multivariate Statistical Methods

**FIGURE 5:**
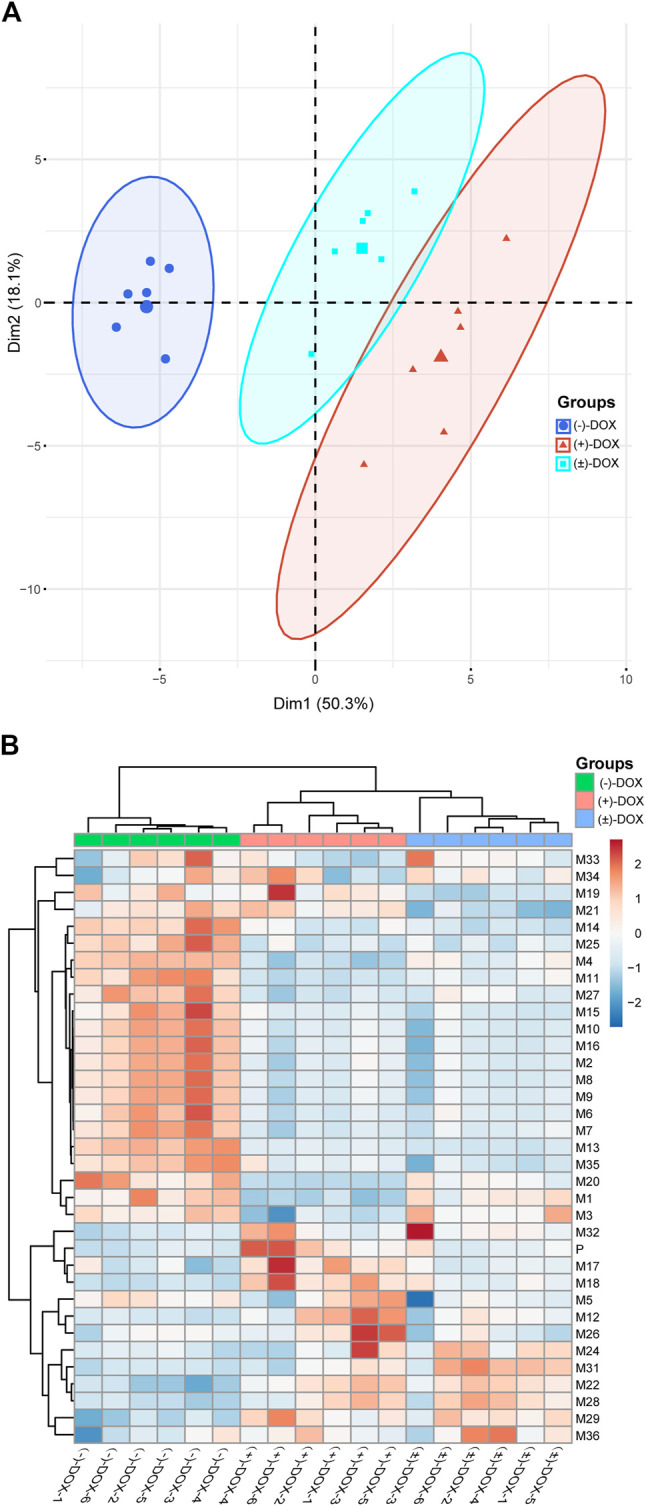
Principal component analysis **(A)** and hierarchical cluster analysis **(B)** of AUCs of the metabolites in rat plasma after intravenous administration of (−)-DOX, (+)-DOX, or (±)-DOX. For heatmap, samples are shown as columns and metabolites arrayed in rows; branch length indicates the degree of variance; and color represents the normalized exposure level of the metabolites.

PCA is an unsupervised technique where knowledge of prior groups is not required. Thus, it is useful to explore the potential grouping of samples in an experiment. To better understand the enantioselectively metabolic profiles of DOX, we tried to interpret the complicated plasma exposure of 34 metabolites obtained from (−)-DOX, (+)-DOX, and (±)-DOX using PCA. As shown in Supplementary Figure S4, PC1 represents the most variant components (50.3%) among all the variant components, and PC2 occupies the other 18.1%. The PCA score plot (Figure 5A) for the two main principal components (PC1 and PC2) revealed a clear difference in the metabolism in rats among (−)-DOX, (+)-DOX, and (±)-DOX. The metabolites in the (−)-DOX group were significantly separated from those in the (+)-DOX and (±)-DOX groups in the PC1 direction (horizontal axis), but the metabolites in the (+)-DOX group and (±)-DOX group were overlapped partly. Therefore, the (−)-DOX metabolism in the rat was definitely different from (+)-DOX, and the (±)-DOX metabolism was more similar to (+)-DOX. As indicated in the 2D PCA loading plot (Supplementary Figure S5), M22, M28, and M31 had a powerful impact on PC1, and they were the main contributors to the significant separation of metabolism between (−)-DOX and (+)-DOX. Furthermore, M21, M15, and M32 were positively correlated with their parent drugs because these vectors were arranged very close to each other. However, M4, M7, and M20 were negatively correlated with their parent drug because of these vectors directing oppositely with parent drug (Supplementary Figure S5).

Based on the HCA analysis (Figure 5B), the samples in (−)-DOX, (+)-DOX, and (±)-DOX groups were clustered together, and the three groups were clearly separated. Moreover, the (+)-DOX and (±)-DOX groups had much more similarity, consistent with the PCA results. In the left dendrogram of Figure 5B, the horizontal direction represents the distance or dissimilarity between metabolites or clusters. It was easy to find that M27, M15, M10, M16, M2, M8, M9, M6, M7, M13, and M35 were arranged closely in one block part in the tree diagram, and these metabolites showed similar metabolic profiles in the (+)-DOX and (±)-DOX groups. Therefore, we could judge that the metabolism of DOX was obviously and complicatedly affected by stereospecificity.

### Chiral Characteristics of Metabolites Containing Chiral Center and Their Relationship With Phases I/II of Metabolism

**FIGURE 6:**
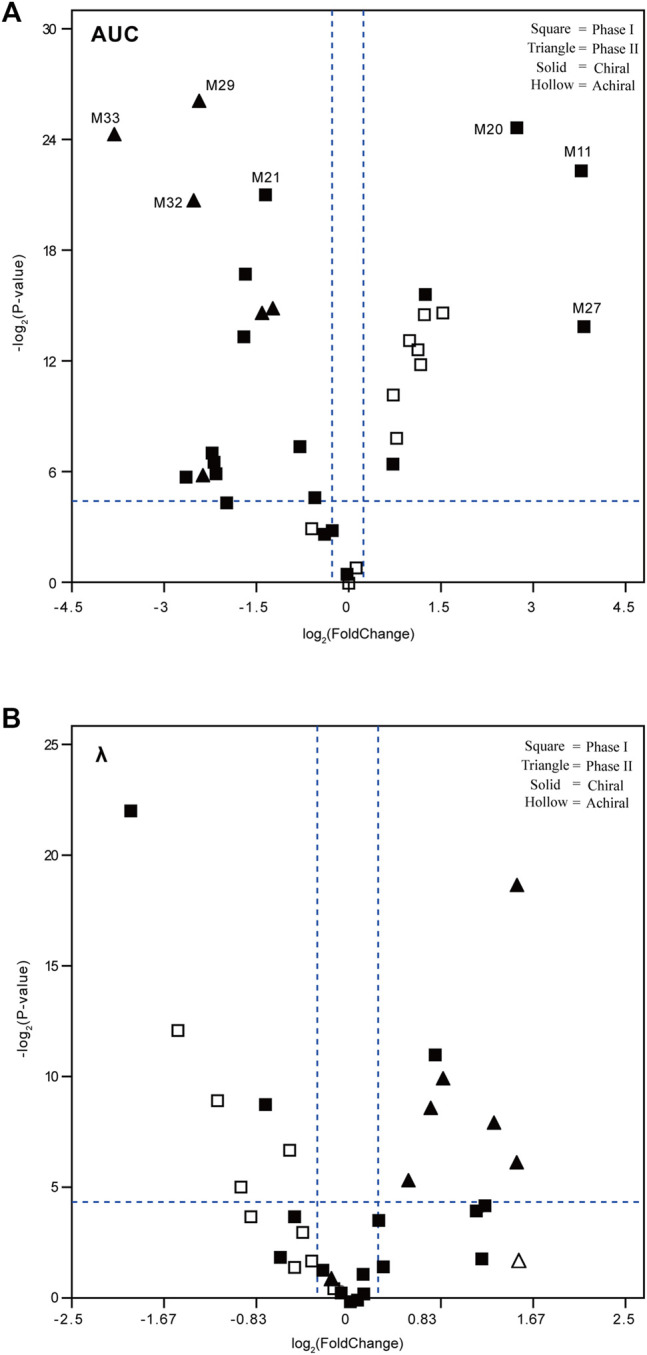
Volcano plots to reveal the exposure level (AUC, **A**) and elimination rate constant (λ, **B**) of the plasma metabolites after intravenous administration of (−)-DOX or (+)-DOX in the rat. The fold change was calculated by dividing the AUC or λ of metabolite derived from (−)-DOX by the AUC or λ of the same metabolite derived from (+)-DOX. The metabolites located in the upper right area or upper left area were separated by two dotted lines (horizontal dotted line: *p* < 0.05; and vertical dotted line: |fold change| > 1.2).

A volcano plot was designed based on the exposure level (AUC) or elimination rate constant (*λ*) ratio value of each metabolite [metabolite [(−)-DOX]/metabolite [(+)-DOX]], in order to reveal metabolic characteristics of the metabolites containing chiral center or not. As shown in the left panel of Figure 6A, the AUC values of 14 metabolites with chiral center metabolized from (−)-DOX, including M21, M29, M32, and M33, were significantly smaller than those from (+)-DOX, and half of them metabolized from (−)-DOX had significantly larger λ values than those from (+)-DOX (Figure 6B, right panel).

In addition, AUC values of the other 12 metabolites from (−)-DOX, including M11, M20, and M27, were significantly higher than those from (+)-DOX (Figure 6A, right panel), and seven of them were achiral compounds. Moreover, the metabolites with statistical significance present in the right panel of Figure 6A and the left panel of Figure 6B were all phase I metabolites. We previously reported that the exposure of (−)-DOX was significantly less than that of (+)-DOX in rat plasma (Li et al., 2015; Liu et al., 2010), and no chiral conversion was observed between (−)-DOX and (+)-DOX at the chiral carbon center (Zhen et al., 2013). Therefore, we speculated that their metabolites with the chiral carbon could not undergo chiral conversion either. Taking these results together, the following explanations should be considered. Firstly, most of the metabolites were definitely produced by chirally metabolic processes in rats. Secondly, among the 34 metabolites from (−)-DOX, 12 metabolites were involved in phase I drug metabolism with much higher plasma concentrations than those derived from (+)-DOX, suggesting that the lower plasma concentration of (−)-DOX in rats was due to its more effective oxidative metabolism. Lastly, with respect to all the seven metabolites with significantly higher elimination rate (*λ*) involved in the (−)-DOX metabolism, six of them belonged to phase II drug metabolism (conjugation reactions), which could be promoted to the faster conversion of (−)-DOX into the easily excreted metabolites.

### Chiral Metabolism by Human or Rat Liver Microsomal Enzymes

**FIGURE 7:**
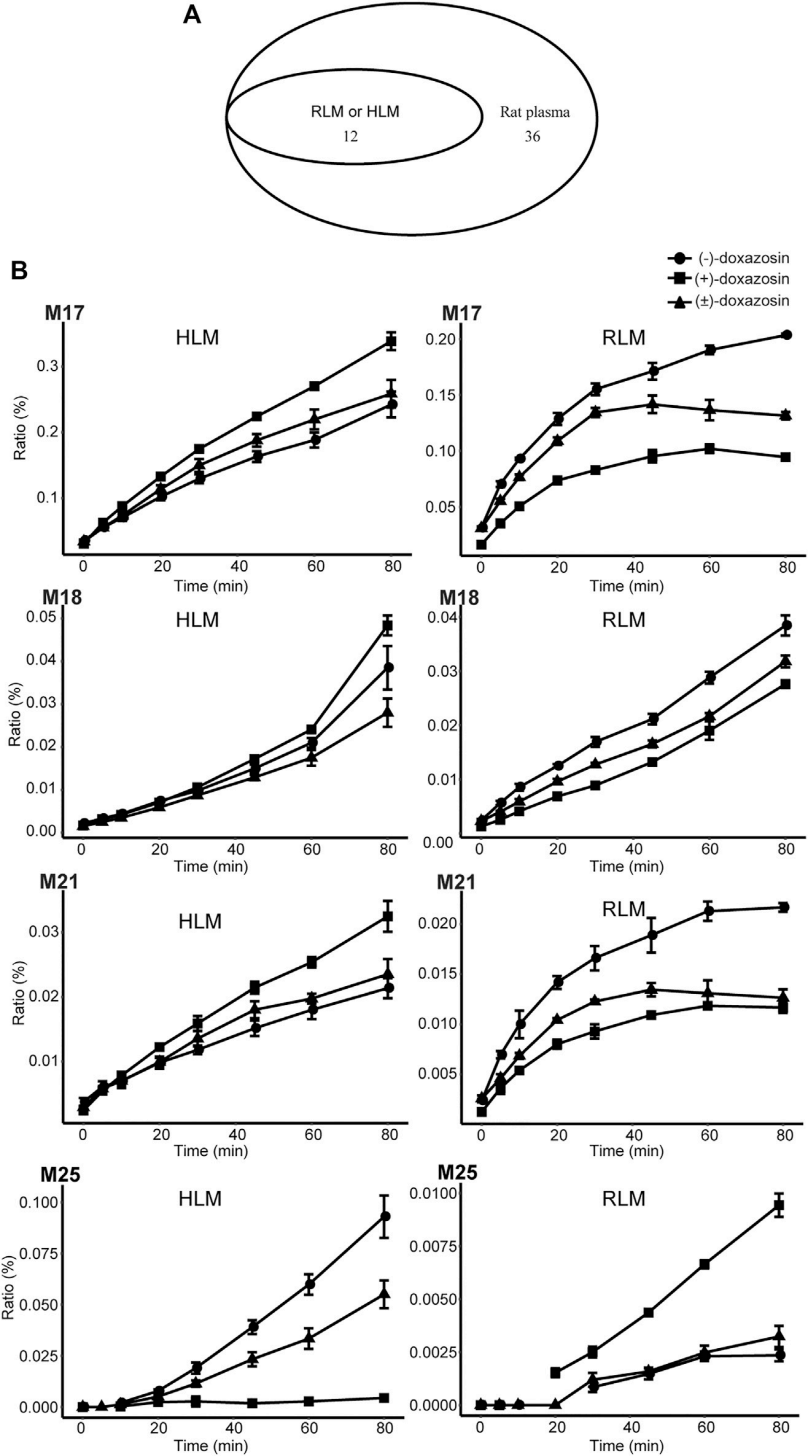
Metabolism of doxazosin by rat and human liver microsomal enzymes. A Venn diagram **(A)** illustrated the comparison among the metabolites found in HLM, RLM, and rat plasma. The chiral metabolic characteristics were opposite for some metabolites (e.g., M17, M18, M21, and M25) between RLM and HLM incubation systems **(B)**. Also, see Supplementary Figure S6.

The mammalian liver, the major site of drug metabolism, contains liver microsomes, especially CYPs, and they are involved mainly in phase I metabolic enzymes. In the present study, we investigated the chiral metabolism of DOX using rat and human liver microsomal enzymes *in vitro* and found 12 metabolites in either RLMs or HLMs (Figure 7A). However, 14 phase I metabolites found in the rat plasma, including M1, M2, M3, M5, M6, M7, M8, M9, M10, M11, M13, M19, M26, and M28, were not found in the liver microsomal system, indicating that other organs, tissues, and non-liver microsomal system of rats were certainly responsible for the 14 phase I metabolites of DOX. Moreover, an in-depth reanalysis of the metabolites of (−)-DOX and (+)-DOX in RLM showed that chiral metabolic characteristics, defined as the differences between the amount of the metabolite metabolized from (−)-isomer and the amount of the same metabolite metabolized from (+)-isomer, of most metabolites (M4, M14, M16, M17, M18, M21, M22, M24, and M25) in RLMs could not represent those in the rat plasma (Table 2 and Table 3). Moreover, chiral metabolic characteristics of M15∼M18, M21, M22, M24, and M25 in RLM were significantly different from those in HLMs (Figure 7B and Supplementary Figure S6), especially for metabolites M17, M18, M21, and M25. Their AUC values were completely opposite between RLMs and HLMs (Figure 7B).

**TABLE 3:** The formation of metabolites (AUC values) for DOX in HLMs and RLMs.

	Human liver microsomes			Rat liver microsomes		
Metabolites	(−)-DOX	(+)-DOX	(±)-DOX	(−)-DOX	(+)-DOX	(±)-DOX
M4	1.348 ± 0.055*^,+^	26.004 ± 0.564^#^	12.182 ± 0.204	7.349 ± 4.218*^,+^	28.439 ± 1.006^#^	15.66 ± 0.177
M14	0.375 ± 0.015	0.369 ± 0.009^#^	0.393 ± 0.011	0.359 ± 0.012	0.341 ± 0.008	0.342 ± 0.013
M15	0.166 ± 0.013^+^	0.169 ± 0.014^#^	0.142 ± 0.004	3.849 ± 0.064*^,^+	8.97 ± 0.303^#^	6.011 ± 0.081
M16	0.036 ± 0.009	NA	0.032 ± 0.005	0.507 ± 0.068*^,+^	11.881 ± 1.387^#^	0.851 ± 0.06
M17	11.697 ± 0.674*^,+^	16.029 ± 0.227^#^	13.162 ± 0.76	12.279 ± 0.299*^,+^	6.516 ± 0.212^#^	9.515 ± 0.342
M18	1.228 ± 0.09*^,+^	1.418 ± 0.036^#^	0.99 ± 0.068	1.639 ± 0.051*^,+^	1.031 ± 0.032^#^	1.268 ± 0.015
M20	143.531 ± 6.46*^,+^	30.895 ± 0.628^#^	68.479 ± 3.732	58.593 ± 2.14*^,+^	37.531 ± 2.08^#^	31.901 ± 0.802
M21	1.094 ± 0.05*^,+^	1.503 ± 0.052^#^	1.208 ± 0.072	1.337 ± 0.059*^,+^	0.746 ± 0.023^#^	0.896 ± 0.039
M22	24.859 ± 1.801*^,+^	21.445 ± 0.83	19.094 ± 1.292	20.511 ± 0.57^+^	19.908 ± 0.793^#^	15.675 ± 0.478
M24	0.225 ± 0.012*	0.305 ± 0.019^#^	0.249 ± 0.019	0.052 ± 0.005^+^	NA	0.03 ± 0.009
M25	2.895 ± 0.25*^,+^	0.183 ± 0.042^#^	1.69 ± 0.224	0.1 ± 0.005*^,+^	0.324 ± 0.012^#^	0.115 ± 0.005
M27	3.586 ± 0.327*	2.833 ± 0.054	3.234 ± 0.312	2.372 ± 0.058*^,+^	0.902 ± 0.131^#^	1.916 ± 0.026

“NA” means it cannot be calculated. Logarithmic transform was performed before statistical analysis. One-way ANOVA followed by Tukey’s HSD test was used. *p < 0.05 versus (+)-DOX; ^+^ *p* < 0.05 versus (±)-DOX; ^#^ *p* < 0.05 versus (±)-DOX.

It seems reasonable to replace HLMs with RLMs to investigate achiral drug metabolism, and both the microsomal systems could partly illustrate the biotransformation of achiral drug metabolism *in vivo*. For example, the same 12 metabolites of DOX existed in HLMs and RLMs, and they were also present in rat plasma. However, once our research involved the concept of chiral metabolism [(−)-DOX and (+)-DOX], the huge difference between HLMs and RLMs in drug metabolism was found. After the application of chiral drugs (−)-DOX and (+)-DOX in the HLMs and RLMs, only 4 of the 12 metabolites were in a similar metabolic pattern. Additionally, most metabolites (8/12) of (−)-DOX and (+)-DOX in the RLMs were considerably different in their metabolic patterns when compared with those in rat plasma, indicating that RLMs could not represent chiral phase I metabolism of DOX in rats.

### Chiral Metabolism by Human CYP Enzymes

The activities of the seven important human CYP enzymes (CYP2E1, CYP2D6, CYP3A4, CYP2C19, CYP1A2, CYP2C8, and CYP2C9) were evaluated by their CYP enzyme-specific substrates (Supplementary Table S1), and their catalytic activities were retained and met the requirements in the following incubation experiments.

**FIGURE 8:**
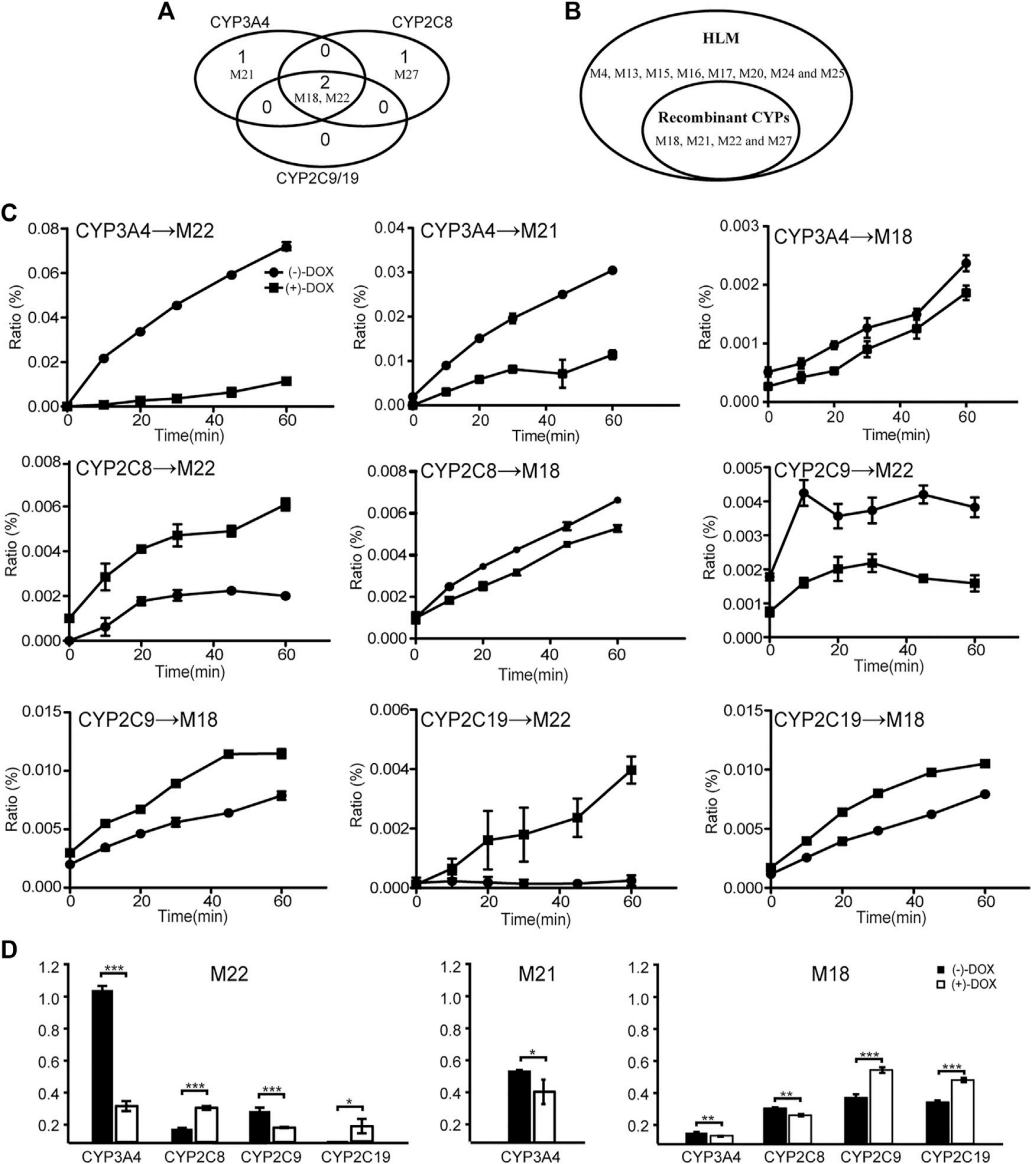
The biotransformation of DOX by CYP enzymes. Four metabolites of DOX were found in CYP3A4, CYP2C8, and CYP2C19 incubation systems. **(A)** A Venn diagram. **(B)** The comparison of metabolites identified between HLM and human CYP enzymes. **(C)** Line charts of metabolites generated from (−)-DOX and (+)-DOX by different human CYP enzymes. **(D)** Summarized AUC values for each metabolite.

Even though 34 metabolites of DOX identified in rat plasma were not found in the CYP2E1, CYP2D6, or CYP1A2 incubation system, two or three metabolites were identified in the CYP3A4, CYP2C19, CYP2C8, or CYP2C9 incubation system (Figure 8A), indicating a crucial role of the four CYPs in the oxidative metabolism of DOX. As shown in Figure 8A, it is easy to find that more than one enzyme participates in the same metabolic reactions, probably due to the poor substrate specificity of CYPs (Guengerich, 2018). Metabolites M4, M13, M15, M16, M17, M20, M24, and M25 found in HLM did not exist in the CYP systems (Figure 8B), suggesting other drug metabolic enzymes in charge of the generation of the eight metabolites in HLM.

As shown in Figure 8C, all the four CYP enzymes stereoselectively catalyzed the formation of corresponding metabolites from DOX (Figure 8C). Among them, CYP3A4 preferentially catalyzed the formation of M18, M21, and M22 from (−)-DOX, while CYP2C19 preferentially catalyzed the formation of M18 and M22 from (+)-DOX (Figure 8C and Figure 8D). In addition, when analyzing the production of metabolites from a relatively quantitative perspective, we found that CYP3A4 (the most abundant drug-metabolizing enzyme in the liver) contributed the most to the oxidative metabolism of DOX. Particularly, the M22 production from (−)-DOX was more than four times that from (+)-DOX. In humans and rats, following oral administration of (±)-DOX, the plasma concentration of the (−)-DOX is lower than that of the (+)-DOX (Liu et al., 2010; Li et al., 2015). When (±)-DOX is incubated with rat liver microsomes, (−)-DOX is depleted much faster than (+)-DOX because of a prominent and stereoselective inhibition of (−)-DOX over (+)-DOX at the CYP3A enzyme (Kong et al., 2015). In this study, we furtherly revealed that the action of CYP3A4 catalyzed the conversion of (−)-DOX to M18, M21, and M22 much stronger than that of (+)-DOX, especially for the conversion of (−)-DOX to M22. Therefore, the hydroxylation of (−)-DOX to M22 catalyzed by CYP3A4 was highly specific and selective.

Unlike 3A4 and 2C19, CYP2C8 preferentially catalyzed (+)-DOX to produce M22 and catalyzed (−)-DOX to produce M18 (Figure 8D). The chiral catalysis of CYP2C9 was just opposite to that of CYP2C8 (Figure 8D). We speculated that the formation of M22 was a hydroxylation reaction, and the formation of M18 was a dehydration reaction after the hydroxylation reaction. Particularly, we considered that it is difficult for the same catalytic center of the same enzyme to have the opposite affinity for (−)-DOX and (+)-DOX. Therefore, we proposed that CYP2C8 or CYP2C9 should contain two active catalytic centers responsible for the formation of M18 and M22, respectively.

Moreover, in comparison with the results obtained from *in vitro* experiments, plasma concentrations of the sum of various (phases I and II) metabolites would reflect the stereoselectivity of DOX and DOX-enantiomers more correctly.

**FIGURE 9:**
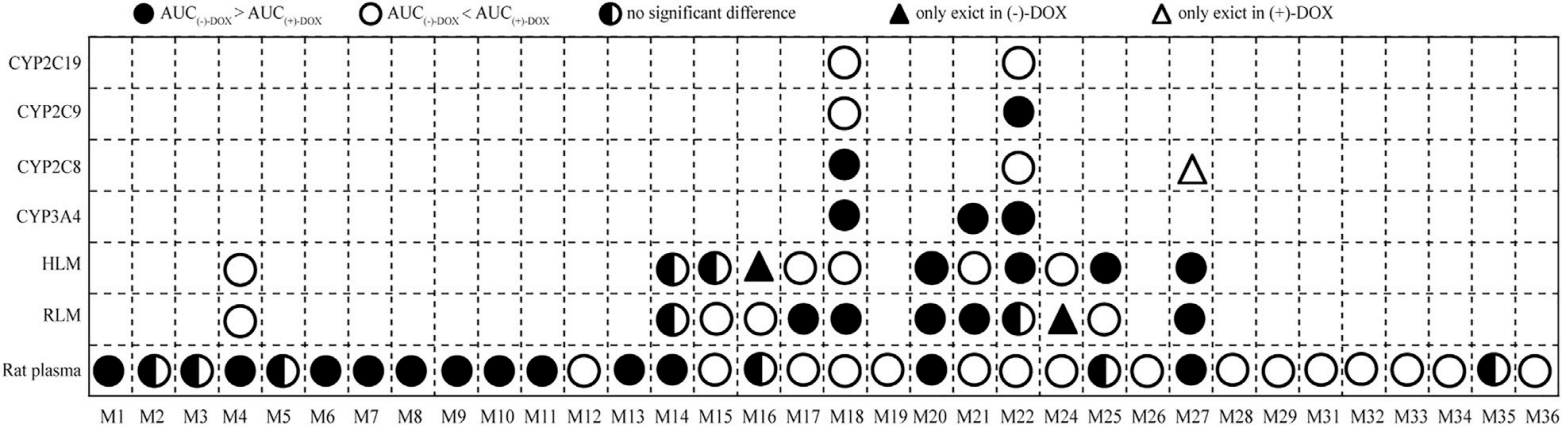
The exposure (AUC0-t) of 34 metabolites indicating the complicated enantioselectivity of the metabolism of DOX *in vivo* and *in vitro* systems after (−)-DOX and (+)-DOX administration.

In summary, we identified 34 metabolites that showed trends over time in rat plasma. After optically pure (−)-DOX, (+)-DOX, and (±)-DOX administration, respectively, we used the multivariate statistical methods to discover the differences of these metabolites based on their exposure and elimination rate and found that the metabolic profile of (±)-DOX was more similar to that of (+)-DOX. The relationship among the metabolites and the most discriminative metabolites between (−)-DOX and (+)-DOX administration were also analyzed. The number of metabolites found in rat plasma was far more than that found in the RLM and HLM systems, indicating that the best way to comprehensively overview the metabolites is *in vivo* rather than *in vitro*. Though the metabolites identified in RLM and HLM were the same, the metabolic profiles of the metabolites from (−)-DOX and (+)-DOX were greatly different, which could be explained by the differences in amount and composition of the metabolic enzymes between RLM and HLM. Furthermore, four CYP enzymes could catalyze DOX to produce metabolites, but their preferences seemed to be different. For example, CYP3A4 highly specifically and selectively catalyzed the formation of M22 from (−)-DOX. The complicated enantioselectivity of the metabolism of DOX *in vivo* and *in vitro* systems, taking the exposure of the metabolites as an example, is summarized and shown in Figure 9.

As a limitation, the present study was not only to find new metabolites but also to see whether there was a chiral metabolic difference in the same metabolite derived from (−)-DOX, (+)-DOX, or (±)-DOX. Therefore, the AUC value of metabolites became the key factor in selecting available metabolites, which inevitably led to missing information on drug metabolites and metabolic pathways. A more detailed analysis of drug metabolites and metabolic pathways needs further investigation. Additionally, we performed a separation using an achiral C18 column in the present study. The enantiomers could not be separated but eluted together to form a single peak. Although optically pure enantiomers were administrated, enantioselective analysis of DOX metabolism in the present study was limited to the chiral center of doxazosin. Enantioselectivity of the metabolites with a new chiral center was unknown, which needs to be studied further. Lastly, as two or three metabolites were identified when doxazosin was incubated with CYP3A4, CYP2C8, CYP2C9, or CYP2C19, it was difficult to simply study the Michaelis–Menten kinetics using recombinant human cytochrome P450 enzymes. We needed to carry out the specifically designed experiments in the near future.

## Conclusion

We established a comprehensive metabolic system using pure optical isomers from *in vivo* to *in vitro*, and the complicated enantioselectivity of the metabolites of DOX was clearly shown either between rats and RLMs/HLMs, between RLMs and HLMs, or between CYP enzymes. Another interesting finding that should be mentioned is that CYP3A4 was a unique enzyme with very high selectivity and activity for (−)-DOX metabolism. More importantly, the comprehensive metabolic system is also suitable to investigate other chiral drugs.

## Data Availability

The original contributions presented in the study are included in the article/Supplementary Material, further inquiries can be directed to the corresponding author.
