# Supplementary material for: Elucidating a Complicated Enantioselective Metabolic Profile: A Study From Rats to Humans Using Optically Pure Doxazosin
Source: Front Pharmacol. 2022 Mar 10;13:834897. doi: 10.3389/fphar.2022.834897 (PMC8960639; doi:10.3389/fphar.2022.834897)
Supplement: Supplementary file 1 [file Image5.pdf]

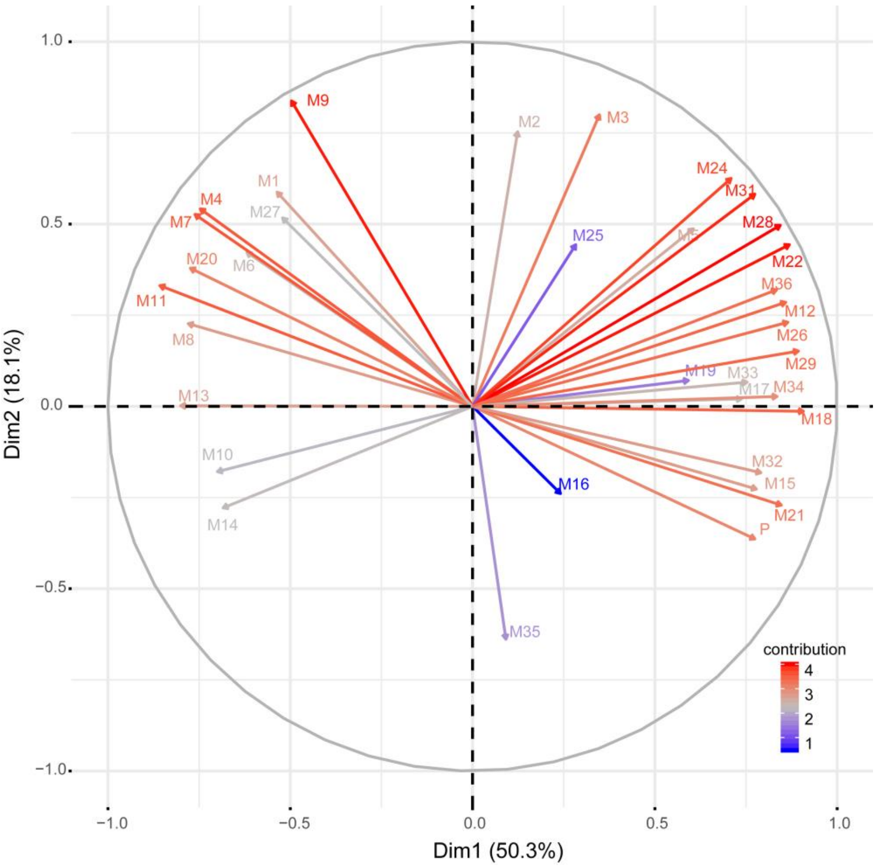

Supplementary Figure S5 PCA loading plot indicating M22, M28 and M31 were the main contributors to the significant separation of metabolism between (-)-DOX and (+)-DOX
