# Supplementary material for: Elucidating a Complicated Enantioselective Metabolic Profile: A Study From Rats to Humans Using Optically Pure Doxazosin
Source: Front Pharmacol. 2022 Mar 10;13:834897. doi: 10.3389/fphar.2022.834897 (PMC8960639; doi:10.3389/fphar.2022.834897)
Supplement: Supplementary file 2 [file Presentation1.pdf]

Supplementary Appendix S1

Elucidation of the Chemical Structures of the Metabolites Except Those Showed in Article.

M3 with a protonated $[M+H]^+$ ion at m/z 288.1470, was eluted at 3.09 min. The *m/z* 245.10497, 221.1053 were found in the MS^2 spectrum. It was 2 Da less than that of M4, showing that there were 2H less than M4 in molecular composition. Therefore, it can be inferred that M3 is a metabolite obtained by oxidation elimination at the 2, 3 or 5, 6 position of the piperazine ring, that the specific reaction site can't be judged from MS^2 spectrum alone (**Table 1** and **Supplementary Figure** S2).

M5 with a protonated $[M+H]^+$ ion at m/z 292.1420 ($C_{13}H_{17}N_5O_3$), was eluted at 2.92 min. The MS^2 spectrum displayed fragment ions at m/z 274.1305, 247.1205, 221.1044. It was 2 Da larger than M4 suggesting that M5 may be a metabolite obtained by an O-demethylation and then monohydroxylation based on M4. The evidence derives from the m/z 274.1305 that corresponds to the M4 loses a methyl group and 2H (**Table 1** and **Supplementary Figure** S2).

M6 with a protonated $[M+H]^+$ ion at m/z 304.1408 was eluted at 3.33 min. The MS^2 of M6 generated the fragment ions at m/z 276.1456, 245.1057, 221.1052. The fragment 276 was 14 Da less than that of 290, and 245 was 2 Da less than that of 247.The elemental composition of $C_{14}H_{17}N_5O_3$ for M6 is an H_2O less than that of M4 ($C_{14}H_{19}N_5O_2$). So, it can be inferred that M6 may be obtained by the elimination of one of the hydroxyl groups after two oxidation reactions of the piperazine ring of M4 (**Table 1** and **Supplementary Figure** S2).

M7, M8 and M10, which were eluted at 3.06, 3.75 and 1.52 min, undergo a series of reactions after the formation of M4. They exhibited protonated $[M+H]^+$ ion at m/z 306.1566 ($C_{14}H_{19}N_5O_3$), 318.1566 ($C_{15}H_{19}N_5O_3$), and 322.1517 ($C_{14}H_{19}N_5O_4$). Different from M7, M8, M10 was formed by the increase of O, CO, 2O occurred at M4, suggested that M7 was formed by a hydroxyl substitution at the piperazine ring of M4; M8 was formed by methylated on the hydroxyl group of the M6 piperazine ring and M10 was formed by two-oxidation reaction on the piperazine ring of M4 (**Table 1** and **Supplementary Figure** S2).

M11 with a protonated $[M+H]^+$ ion at m/z 332.1740 ($C_{16}H_{21}N_5O_3$), which was eluted at 3.93 min. MS^2 gave the base peak of m/z 290.1617, 247.1194. The product ion at m/z 290.1631, 247.1205 indicated an unchanged amino-dimethoxy quinazoline piperazine system. Therefore, M11 was tentatively identified as metabolite was formed by the cracking of the DOX in the 1, 4-benzenedioxy. The structures of M13-M18 were easy to elucidated and shown as in **Table 1** and **Supplementary Figure** S2.

M26 with a protonated $[M+H]^+$ ion at m/z 480.1907, which was eluted at 3.37 min. The MS^2 of M26 generated the fragment ion at m/z 318.1580, 290.1630, 247.1209. The ion at m/z 480 was 28 Da higher than that of DOX, showing that there was a CO more than DOX in molecular composition. So, we concluded that M26 was produced by O-methylation after oxidation reaction of DOX and elimination the hydroxyl groups after oxidation reactions at 2 or 3 position of the 1, 4-benzodioxane (**Table 1** and **Supplementary Figure** S2).

M29 and M30 with same protonated $[M+H]^+$ ion at m/z 548.1482, which were eluted at 2.91 and 3.08 min, 80 Da larger than for M19-M23 suggesting a sulfate conjugation. MS^2 gave the base peak of m/z 468.1907, 344.1723. The fragmentation behaviour of m/z 468.1690 was proposed by losing SO_3. So, we infer that M29 and M30 were the metabolites produced by DOX undergoing oxidation reaction and then combined with SO_3 (**Table 1**).

M31 was eluted at 3.07 min and showed a protonated $[M+H]^+$ at m/z 593.2061, 107 Da larger than m/z 468 (M19-M23) suggesting a taurine conjugation. M32 and M33 with identical protonated $[M+H]^+$ ion at m/z 614.2109, and they were occurred a glucuronide-binding reaction after the 6 or 7 position of DOX quinazoline O-demethylation. M34 with a protonated $[M+H]^+$ ion at m/z 628.22644; it was 176 Da higher than DOX, indicating that they were the glucosinolate conjugate of DOX. MS^2 gave the base peak of m/z 452.1939 via loss of a glucuronide moiety. Similarly, M35 and M36 were the doxazosin-O-glucuronide metabolite (**Table 1**).
