# Supplementary figures and images for: Elucidating a Complicated Enantioselective Metabolic Profile: A Study From Rats to Humans Using Optically Pure Doxazosin
Source: Front Pharmacol. 2022 Mar 10;13:834897. doi: 10.3389/fphar.2022.834897 (PMC8960639; doi:10.3389/fphar.2022.834897)

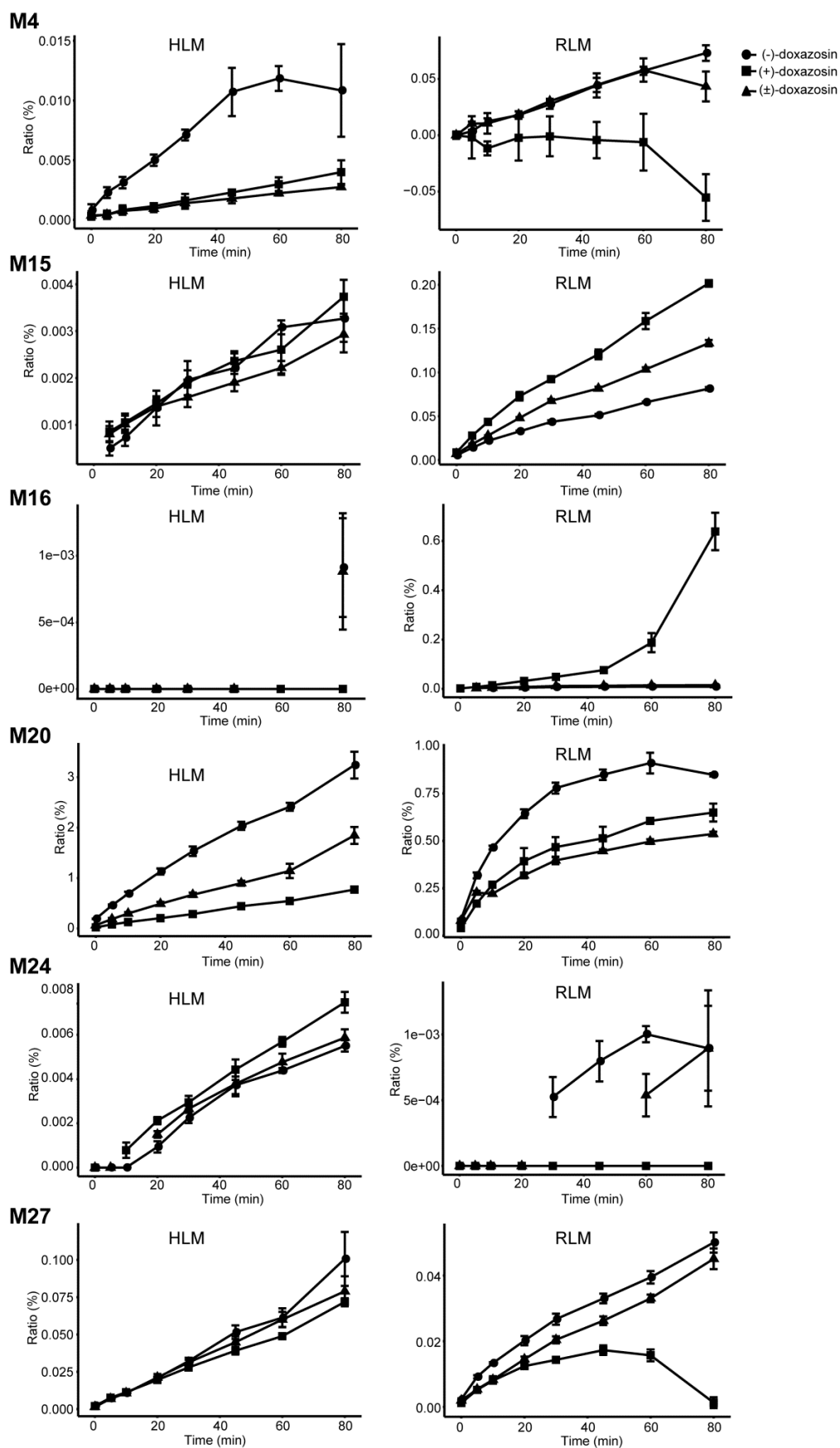

**Supplementary Figure S6** Chiral metabolic characteristics of M14~M16, M20, M24 and M27 in RLMs and in HLMs

Supplement: Supplementary file 3 [file Image6.pdf]

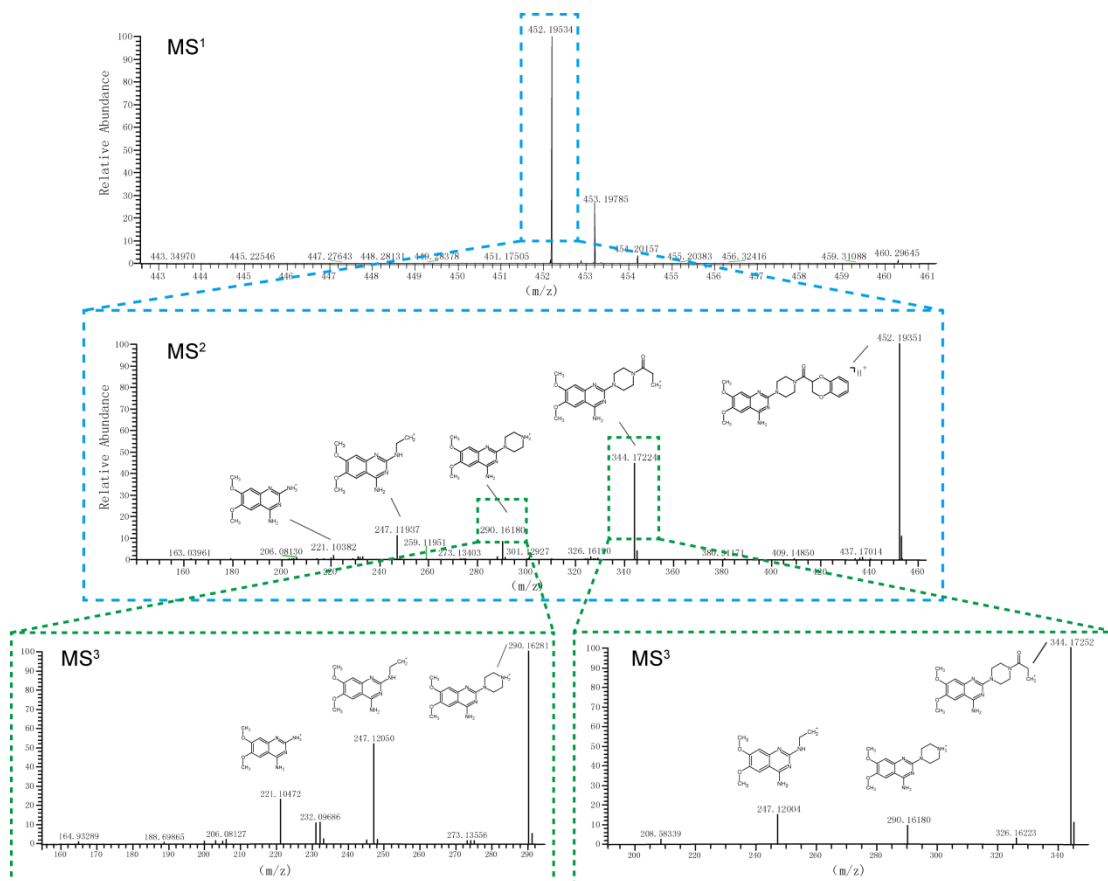

**Supplementary Figure S1** Illustrations of the fragmentation patterns of DOX in ESI-HCD-MS

Supplement: Supplementary file 7 [file Image1.pdf]
