# Supplementary material for: Elucidating a Complicated Enantioselective Metabolic Profile: A Study From Rats to Humans Using Optically Pure Doxazosin
Source: Front Pharmacol. 2022 Mar 10;13:834897. doi: 10.3389/fphar.2022.834897 (PMC8960639; doi:10.3389/fphar.2022.834897)
Supplement: Supplementary file 4 [file Image4.pdf]

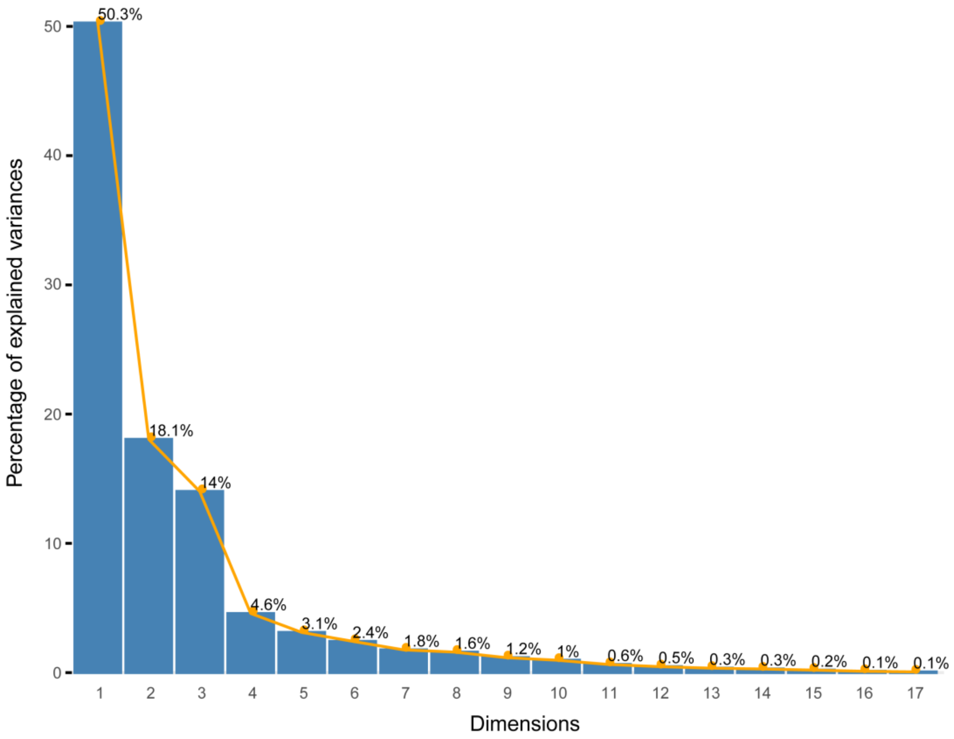

Supplementary Figure S4 Scree plot for the PCA analysis indicating the major variant components
