# Supplementary material for: Elucidating a Complicated Enantioselective Metabolic Profile: A Study From Rats to Humans Using Optically Pure Doxazosin
Source: Front Pharmacol. 2022 Mar 10;13:834897. doi: 10.3389/fphar.2022.834897 (PMC8960639; doi:10.3389/fphar.2022.834897)
Supplement: Supplementary file 5 [file Image2.pdf]

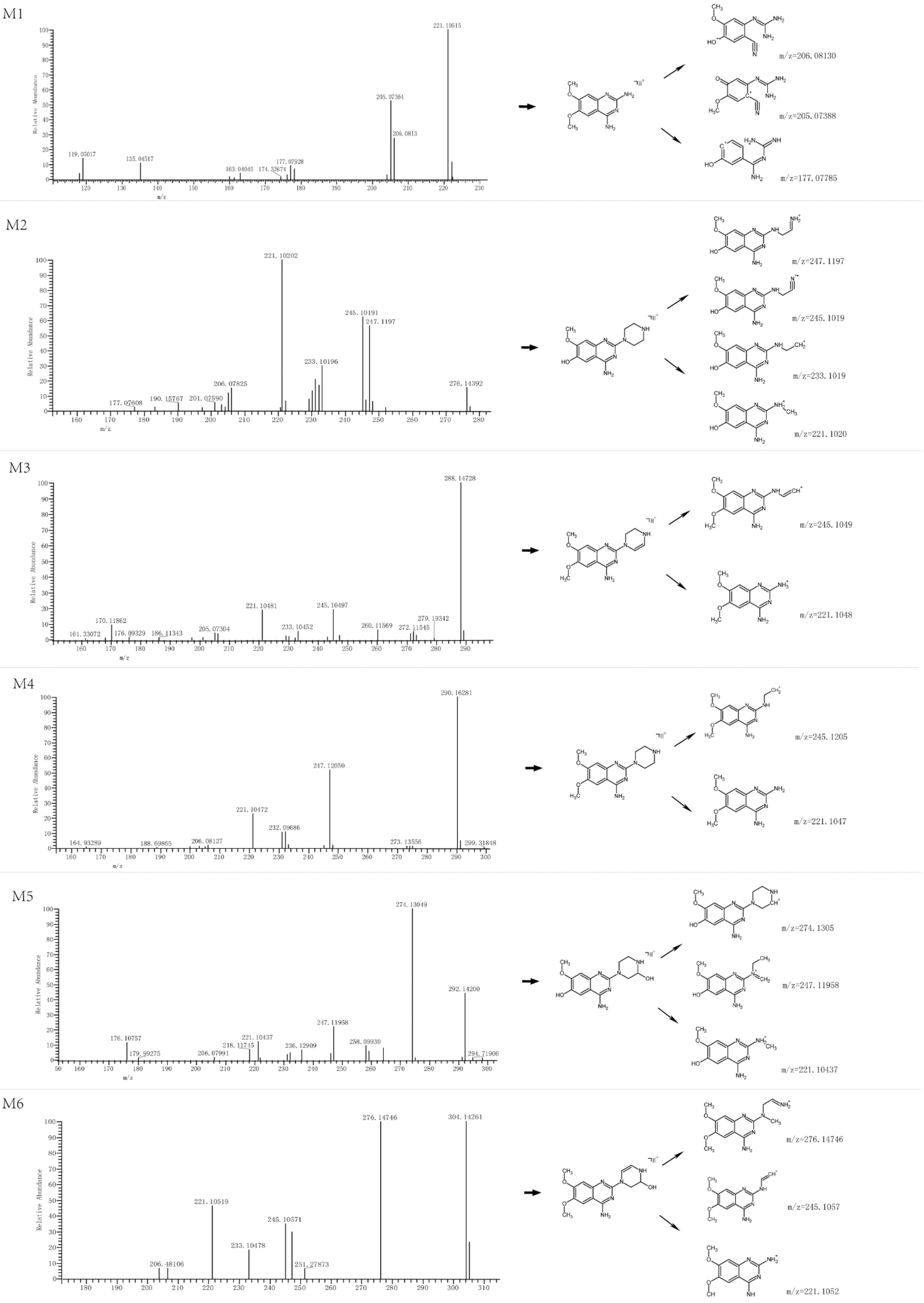

Supplementary Figure S2 MS/MS spectra of metabolites and the chemical structures of major fragment ions

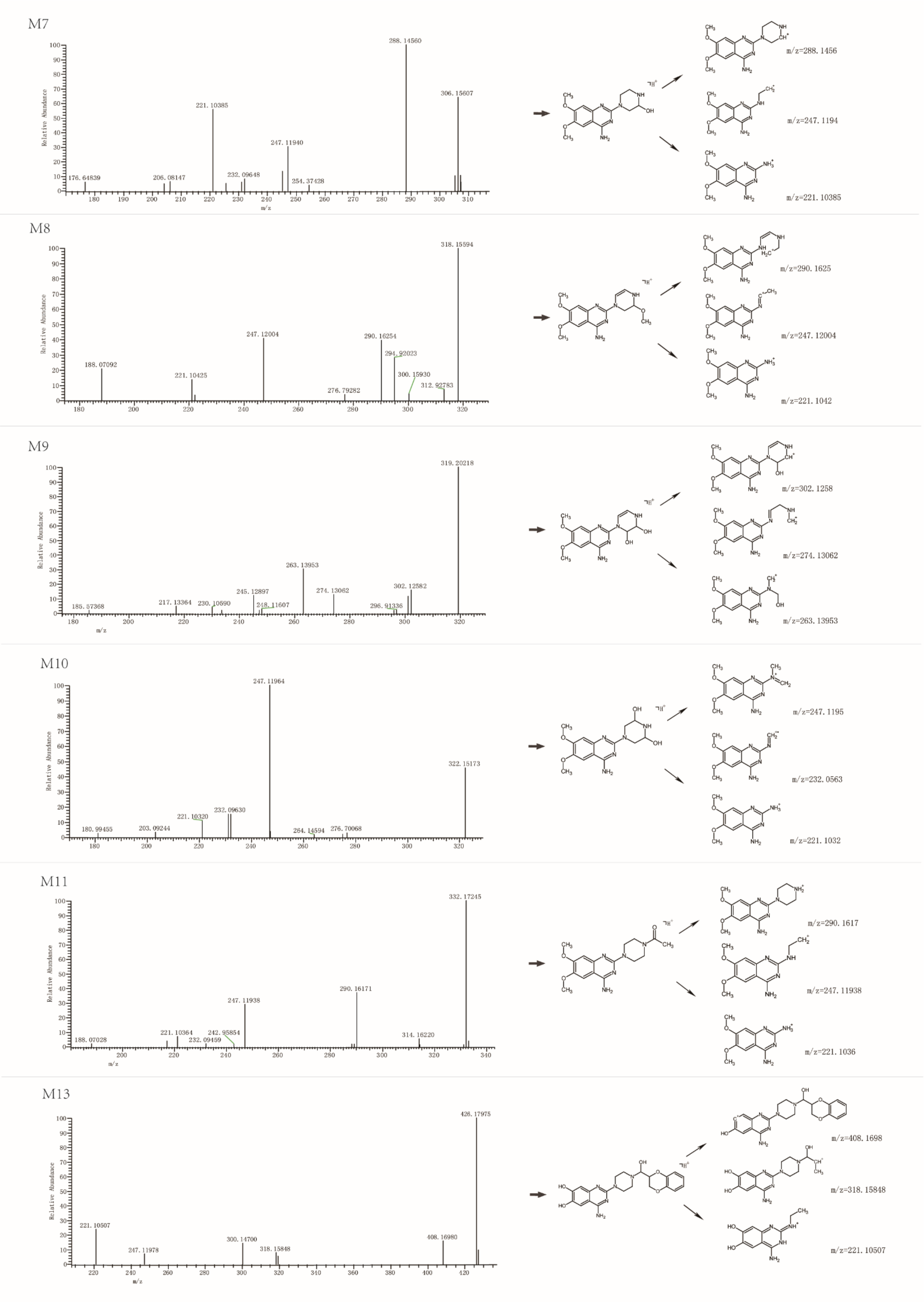

Supplementary Figure S2 (Continued) MS/MS spectra of metabolites and the chemical structures of major fragment ions

M14

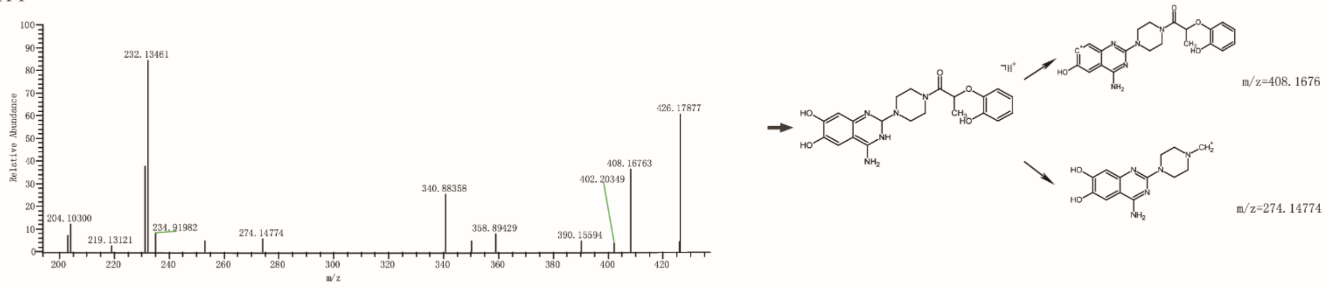

M15

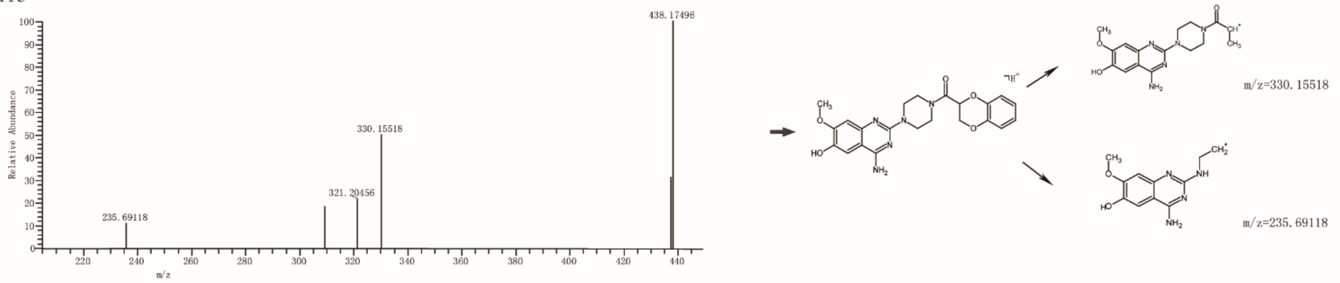

M16

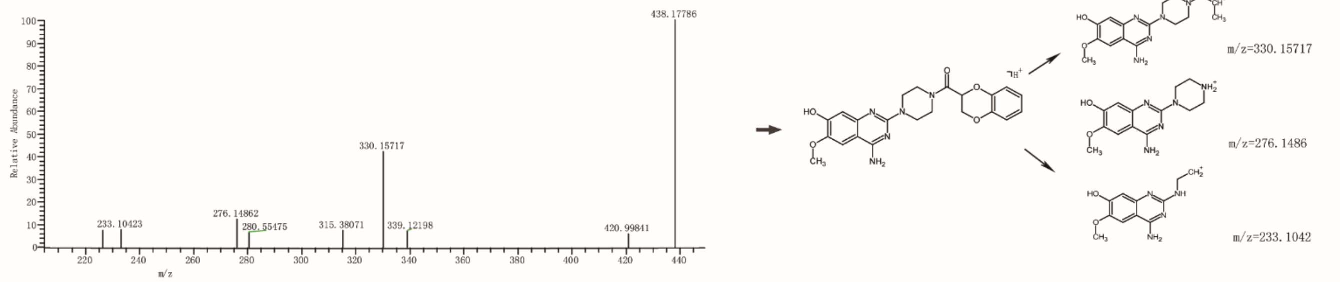

M17

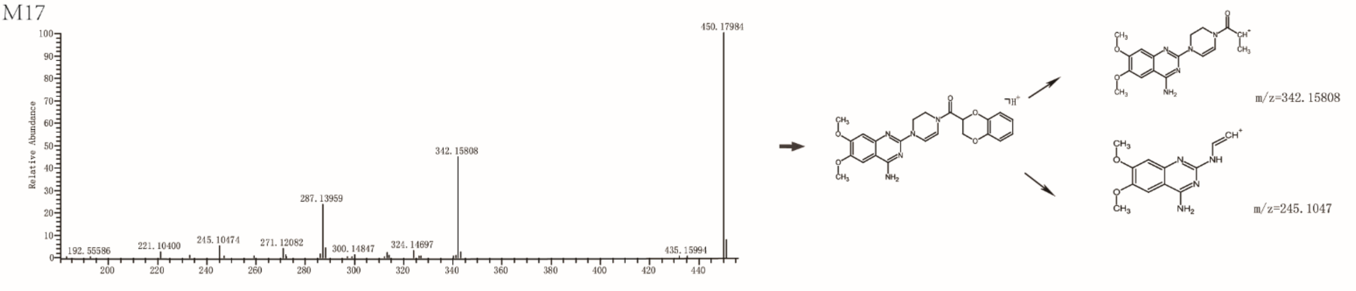

M18

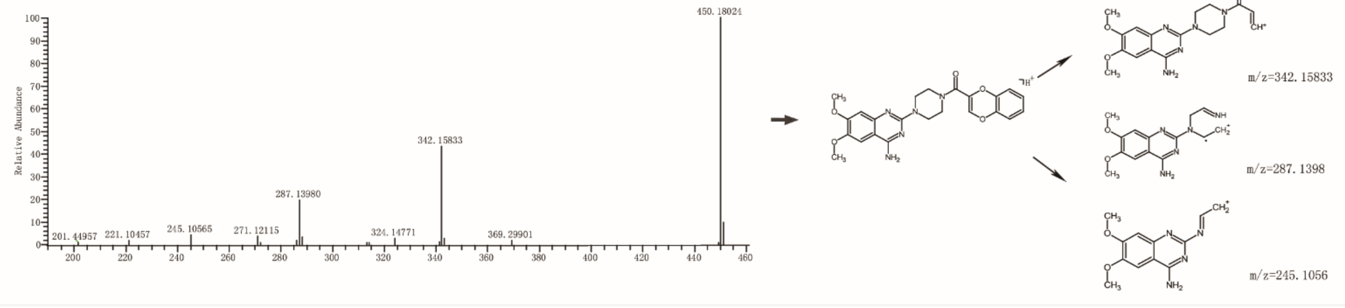

M19

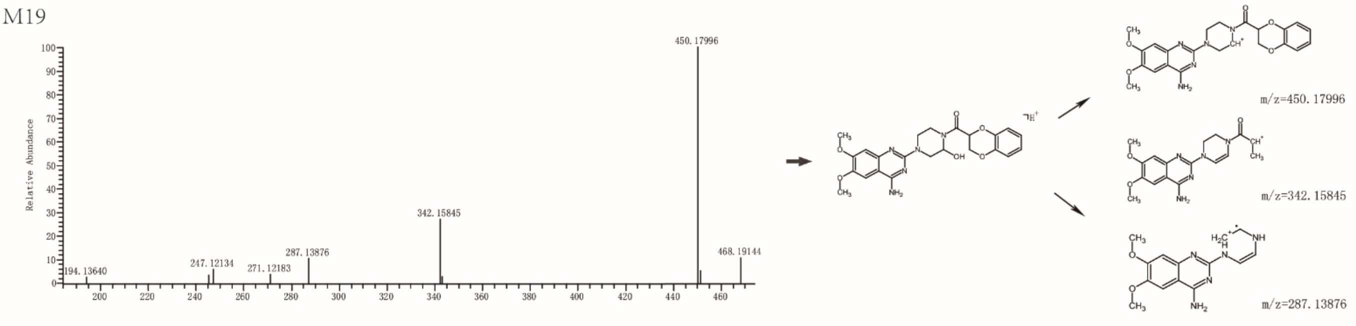

Supplementary Figure S2 (Continued) MS/MS spectra of metabolites and the chemical structures of major fragment ions

M20

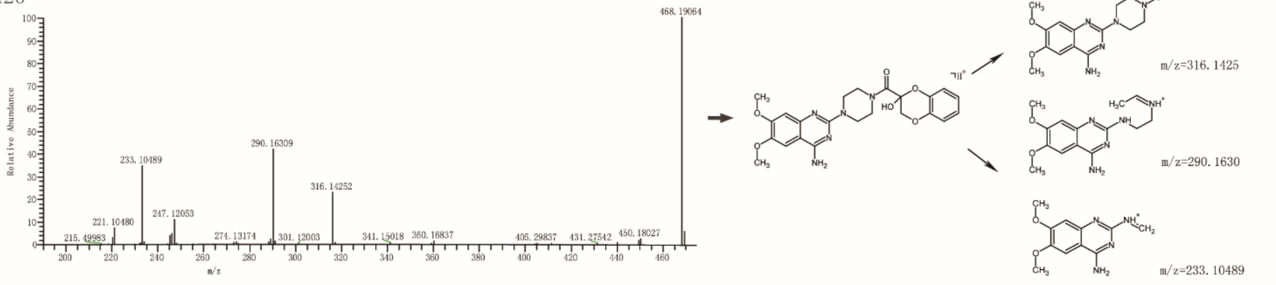

M21

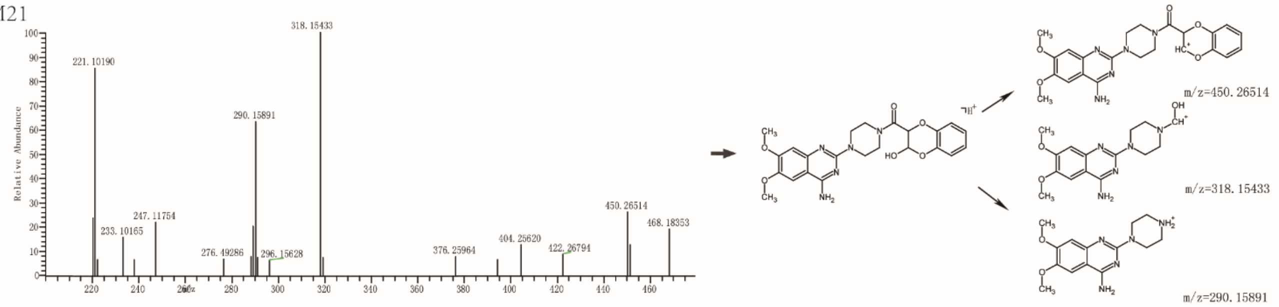

M22

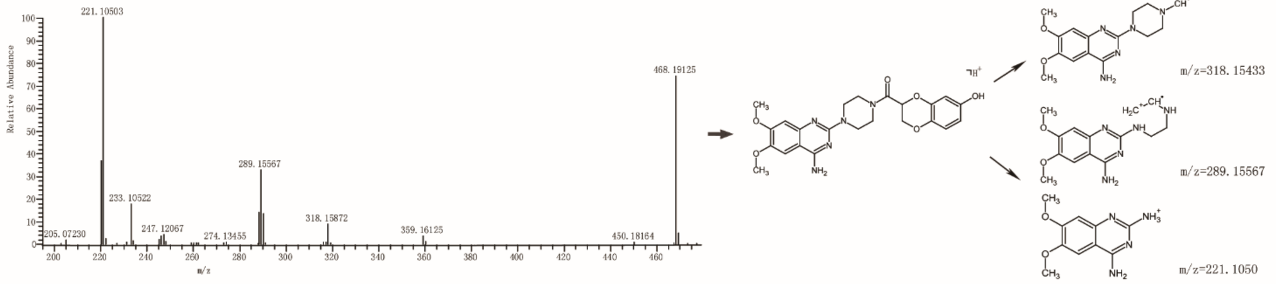

M23

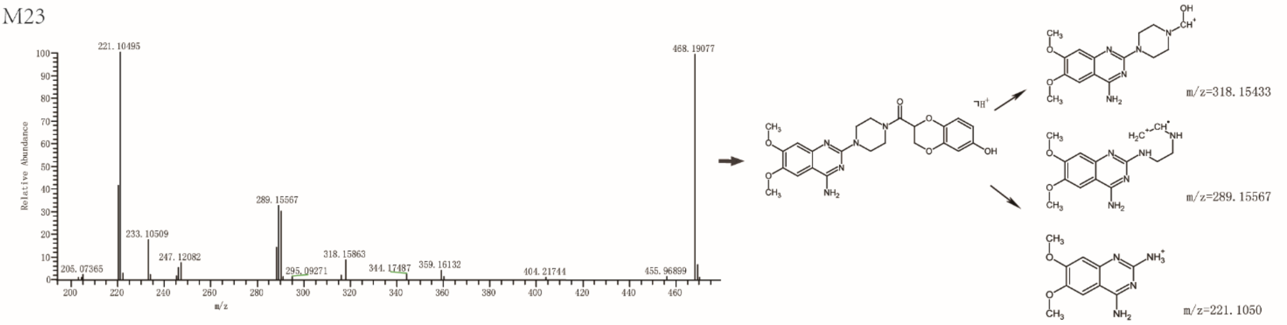

M24

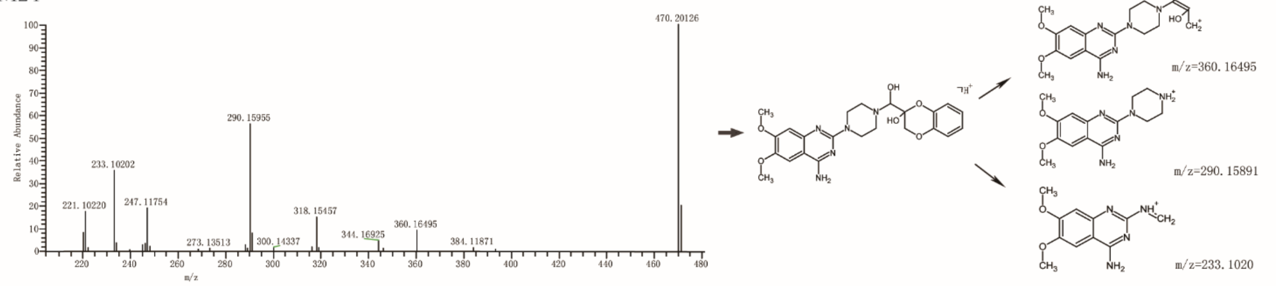

M25

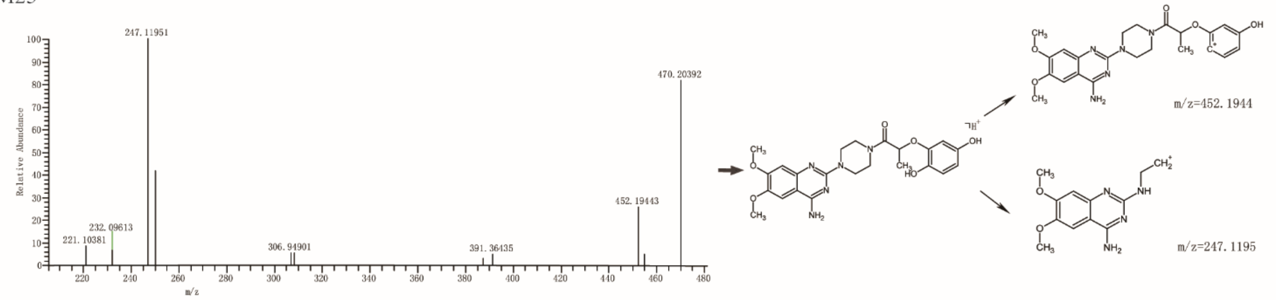

Supplementary Figure S2 (Continued) MS/MS spectra of metabolites and the chemical structures of major fragment ions

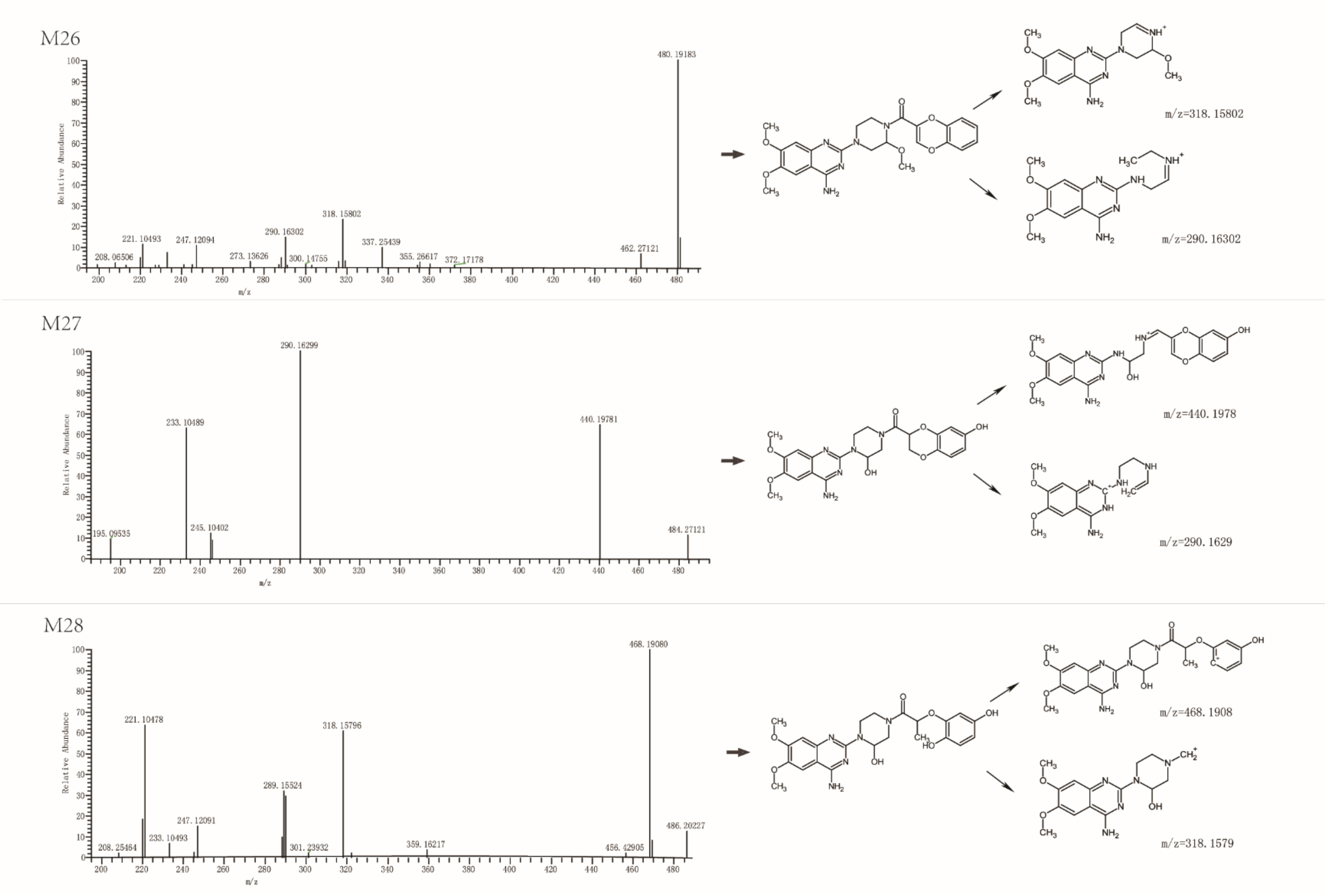

Supplementary Figure S2 (Continued) MS/MS spectra of metabolites and the chemical structures of major fragment ions
