# Supplementary material for: Elucidating a Complicated Enantioselective Metabolic Profile: A Study From Rats to Humans Using Optically Pure Doxazosin
Source: Front Pharmacol. 2022 Mar 10;13:834897. doi: 10.3389/fphar.2022.834897 (PMC8960639; doi:10.3389/fphar.2022.834897)
Supplement: Supplementary file 6 [file Image3.pdf]

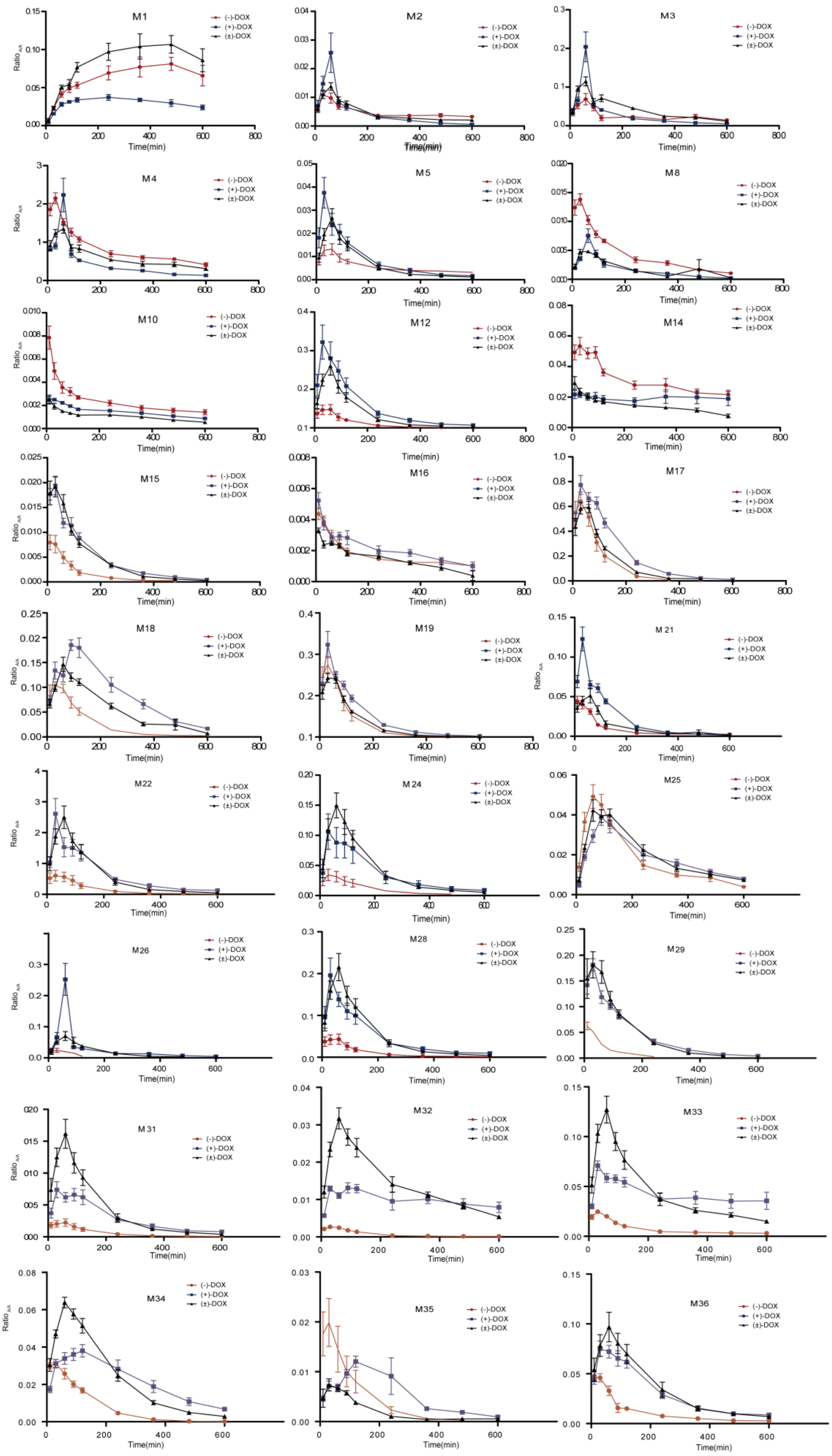

Supplementary Figure S3 The mean plasma concentration-time curves for the metabolites of DOX obtained from six rats after intravenous injection of (-)-DOX, (+)-DOX or (±)-DOX, respectively.
